# Functional dissection of PRC1 subunits RYBP and YAF2 during neural differentiation of embryonic stem cells

**DOI:** 10.1038/s41467-023-42507-9

**Published:** 2023-11-07

**Authors:** Yanjiang Liu, Gongcheng Hu, Shengxiong Yang, Mingze Yao, Zicong Liu, Chenghong Yan, Yulin Wen, Wangfang Ping, Juehan Wang, Yawei Song, Xiaotao Dong, Guangjin Pan, Hongjie Yao

**Affiliations:** 1 State Key Laboratory of Respiratory Disease, The First Affiliated Hospital of Guangzhou Medical University, Joint School of Life Sciences, Guangzhou Institutes of Biomedicine and Health, Chinese Academy of Sciences, Guangzhou Medical University, Guangzhou, China; 2 Department of Basic Research, Guangzhou National Laboratory, Guangzhou, China; 3 University of Chinese Academy of Sciences, Beijing, China

**Keywords:** Embryonic stem cells, Gene silencing, Epigenetics

## Abstract

Polycomb repressive complex 1 (PRC1) comprises two different complexes: CBX-containing canonical PRC1 (cPRC1) and RYBP/YAF2-containing variant PRC1 (vPRC1). RYBP-vPRC1 or YAF2-vPRC1 catalyzes H2AK119ub through a positive-feedback model; however, whether RYBP and YAF2 have different regulatory functions is still unclear. Here, we show that the expression of RYBP and YAF2 decreases and increases, respectively, during neural differentiation of embryonic stem cells (ESCs). *Rybp* knockout impairs neural differentiation by activating Wnt signaling and derepressing nonneuroectoderm-associated genes. However, *Yaf2* knockout promotes neural differentiation and leads to redistribution of RYBP binding, increases enrichment of RYBP and H2AK119ub on the RYBP-YAF2 cotargeted genes, and prevents ectopic derepression of nonneuroectoderm-associated genes in neural-differentiated cells. Taken together, this study reveals that RYBP and YAF2 function differentially in regulating mESC neural differentiation.

## Abstract (web summary)

RYBP and YAF2 are a pair of paralog subunits of variant PRC1. Here, authors show that RYBP decrease but YAF2 increase during mESC neural differentiation, and that they are not redundant but function differentially in regulating this differentiation.

## Introduction

Polycomb group (PcG) proteins are generally enriched in facultative heterochromatin^1^ and behave as vital epigenetic regulators of transcriptional repression, with key roles in multiple biological processes, including pluripotency, differentiation and disease^2–6^. PcG proteins typically assemble into one of two large multiprotein complexes: Polycomb repressive complex 1 (PRC1) and Polycomb repressive complex 2 (PRC2)^7^. PRC1 monoubiquitylates histone H2A at Lys119 (H2AK119ub)^8,9^, whereas PRC2 methylates histone H3 at Lys27 (H3K27me2/3)^10–12^.

PRC1 is categorized as either cPRC1 or vPRC1^7,13–15^. In addition to the catalytic core (RING1B or its paralog RING1A) of PRC1, cPRC1 complexes assemble either PCGF2 or PCGF4 and include one of five chromodomain-containing paralogs (CBX2, CBX4, CBX6, CBX7 or CBX8) and a polyhomeotic (PHC) subunit (PHC1, PHC2 or PHC3)^3^. While the recruitment of PRC1 in vertebrates is still a debated topic, emerging evidence has shown that PRC1 can be recruited to target sites through sequence-specific DNA-binding factors, chromatin-associated RNAs and CpG islands (CGIs)^7,16^. cPRC1 is recruited by H3K27me3 through the CBX protein, resulting in chromatin compaction and gene repression^17–20^.

Unlike cPRC1, vPRC1 complexes can assemble any of the six PCGF proteins (PCGF1-6) and include RING1A/B and YY1-binding protein (RYBP) (or its paralog YAF2) as well as various additional subunits depending on the PCGF component present in the complex^13,14^. vPRC1 exhibits strong catalytic activity and is recruited to its targets independent of PRC2 and H3K27me3^13,14,21–24^.

RYBP and YAF2 share very high homology in the amino-terminus containing zinc finger motifs and moderate similarity in the carboxyl-terminus; RYBP also contains a unique region in the middle of the protein that is absent in YAF2^25–27^. RYBP and YAF2 dramatically stimulate the E3 ubiquitin ligase activity of PRC1^22^, and they catalyze ubiquitination of H2A on neighboring nucleosomes by recruiting RYBP-PRC1 or YAF2-PRC1 complexes through a positive feedback model^28^. A key unanswered question is whether RYBP and YAF2 are functionally redundant or play mechanistically diverse roles.

In this study, we used mESC neural differentiation as a model system and discovered opposite expression trends for *Rybp* and *Yaf2* (decreases in *Rybp* but increases in *Yaf2*) during mESC neural differentiation. Moreover, the absence of either *Rybp* or *Yaf2* led to changes in the efficiency of mESC neural differentiation, with *Rybp* loss reducing but *Yaf2* loss increasing efficiency. Loss of *Rybp* or *Yaf2* affects distinct targets within differentially expressed genes and H2AK119ub enrichment. Together, our results suggested that RYBP-PRC1 and YAF2-PRC1 are not functionally redundant but contribute to precise vPRC1 regulation during the neural differentiation of mESCs.

## Results

### RYBP and YAF2 competitively bind to RING1B

As RYBP and its paralog YAF2 have been identified as variant PRC1 subunits^13,14,29^, we sought to determine which domains are critical for RYBP/YAF2 associated with RING1B. To this end, we performed domain mapping of proteins between RING1B and RYBP or YAF2. To explore the domain in RING1B required for interaction with RYBP or YAF2, we transfected Flag-tagged full-length *Ring1b*, *Ring1b*-N-terminus (aa 1-50), *Ring1b*-ring finger region (aa 49-95), and *Ring1b*-C-terminus (aa 95-336) constructs into HEK293T cells, respectively, and then performed Flag co-IP experiments to test their binding to either RYBP or YAF2 (Supplementary Fig. 1a). Full-length RING1B and the deletion containing the C-terminus (aa 95-336) interacted with RYBP or YAF2, whereas the N-terminus (aa 1-50) and ring finger region (aa 49-95) of RING1B did not (Supplementary Fig. 1b, c).

We further examined the domains in RYBP required for the interaction with RING1B. Full-length RYBP, RYBP^Δzinc finger^ (aa 45-228), and RYBP^ΔC-terminus^ (aa 1-145) were tested for their binding to RING1B (Supplementary Fig. 1d). Our data indicated that full-length RYBP and the deletion lacking the zinc finger (aa 45-228) were able to interact with RING1B but that the deletion lacking the C-terminus (aa 1-145) was not (Supplementary Fig. 1e). Furthermore, to investigate the domains of YAF2 responsible for binding to RING1B, we transfected Flag-tagged full-length *Yaf2*, *Yaf2*^Δzinc finger^ (aa 44-179) and *Yaf2*^ΔC-terminus^ (aa 1-101) constructs into HEK293T cells, respectively, and performed FLAG co-IP experiments (Supplementary Fig. 1f). Full-length YAF2 and the YAF2 deletion without the zinc finger (aa 44-179) interacted with RING1B, but the YAF2 deletion without the C-terminus (aa: 1-101) did not (Supplementary Fig. 1g). The above data indicated that the C-terminus of RYBP and YAF2 interact with the C-terminus of RING1B.

**Fig. 1:**
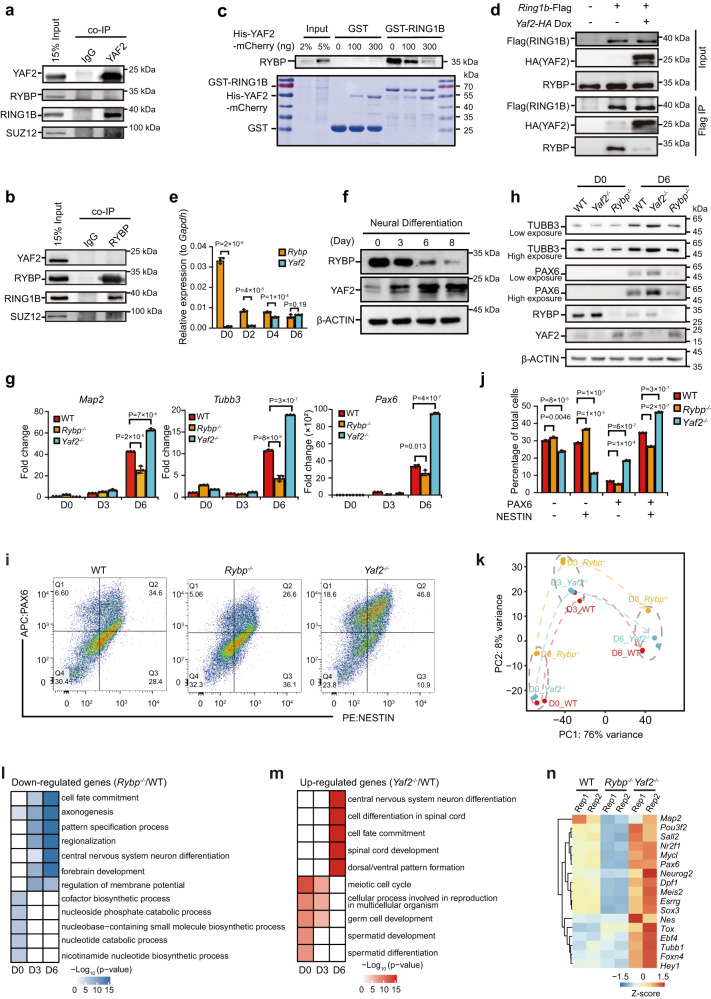
RYBP competes with YAF2 in binding to RING1B, and *Rybp*^−/−^ inhibits but *Yaf2*^−/−^ promotes neural differentiation of mESCs. **a**, **b** Detection of the interaction between PRC1/2 subunits and YAF2 (**a**) or RYBP (**b**) by co-IP in HEK293T cells. **c** GST pull-down assay to detect the interaction between RYBP/YAF2 and GST-RING1B in mESCs. Upper: Western blot analysis for RYBP after GST pull-down; Bottom: Coomassie blue staining of purified GST, GST-RING1B, and increasing amounts of His-YAF2 together with total cell extracts of mESCs before GST pull-down. **d** Detection of the interaction between RYBP/YAF2 and RING1B with or without Dox-inducible HA-YAF2 by Flag co-IP in mESCs. **e** RT-qPCR analysis of *Rybp* or *Yaf2* expression during mESC neural differentiation from day 0 to day 6. **f** Western blot analysis of RYBP and YAF2 expression during mESC neural differentiation from day 0 to day 8. **g** RT-qPCR analysis of *Map2*, *Tubb3* and *Pax6* expression during neural differentiation of wild-type, *Rybp*^−/−^ and *Yaf2*^−/−^ mESCs. The results are shown relative to the wild-type on day 0. **h** Western blot analysis of TUBB3, PAX6, RYBP and YAF2 expression during neural differentiation of wild-type, *Rybp*^−/−^ and *Yaf2*^−/−^ mESCs. **i** FACS data for NESTIN and PAX6 expression in neural-differentiated cells on day 6. **j** Statistical analysis of the cells described in panel (**i**). **k** PCA of RNA-seq in wild-type, *Rybp*^−/−^ and *Yaf2*^−/−^ cells during neural differentiation of mESCs. **l** GO analysis of downregulated genes in *Rybp*^−/−^ cells relative to wild-type mESCs during neural differentiation. **m** GO analysis of upregulated genes in *Yaf2*^−/−^ cells relative to wild-type mESCs during neural differentiation. **n** Heatmap illustrating the expression pattern of neural marker genes that are shown as row Z scores in neural differentiated wild-type, *Rybp*^−/−^ and *Yaf2*^−/−^ cells on day 6. For **a**–**d,** **f** and **h**, Representative immunoblots (*n* = 3 independent experiments) are shown. For **e,** **g** and **j**, data are represented as the mean values ± s.d.s with the indicated significance from two-sided *t* test, *n* = 3 independent experiments. For **l** and **m**, significance was calculated by hypergeometric distribution. Source data are provided as a Source Data file.

To better decipher the relationship between RYBP-RING1B and YAF2-RING1B, we performed endogenous coimmunoprecipitation (co-IP) experiments using either an anti-YAF2 antibody or an anti-RYBP antibody. Co-IP experiments indicated that the YAF2 complex contains RING1B without RYBP (Fig. 1a). Reciprocally, the anti-RYBP antibody coprecipitated endogenous RING1B but not YAF2 (Fig. 1b). These data show that RING1B forms separate protein complexes containing either RYBP or YAF2 in HEK293T cells, suggesting that RING1B may acquire specific functions by interacting with either RYBP or YAF2 under specific conditions.

Because the C-terminus of YAF2 or RYBP interacts with RING1B, and YAF2 or RYBP form independent protein complexes with PRC1, we evaluated whether YAF2 competes with RYBP for binding to RING1B. To test this hypothesis in vitro, we purified glutathione-S-transferase (GST), GST-RING1B, and His-YAF2 fusion proteins expressed in *E. coli* BL21 (DE3) and performed a GST pulldown assay. The gradually increasing amount of in vitro purified His-YAF2 fusion protein prevented the interaction of GST-RING1B with endogenous RYBP in total cell extracts (TCEs) from mESCs (Fig. 1c). To verify whether YAF2 competes with RYBP in interacting with RING1B in vivo, we transfected the *Ring1b*-Flag construct into doxycycline-inducible *Yaf2*-HA stable cell lines. Our data indicated that inducing YAF2-HA expression prevented the interaction between FLAG-RING1B and RYBP in mESCs (Fig. 1d).

Taken together, our data indicate that the vPRC1 subunits RYBP and YAF2 interact with the C-terminus of RING1B in a competitive manner.

### RYBP is downregulated but YAF2 is upregulated during neural differentiation of mESCs

By using RNA-seq data from our recent study^4^, we found that the *Rybp* expression level in mESCs was much higher than that in neural progenitor cells (NPCs) (Supplementary Fig. 2a). In contrast, the *Yaf2* expression level in mESCs was much lower than that in NPCs (Supplementary Fig. 2b). We then induced the differentiation of mESCs toward a neural fate by using N2B27 medium and examined the expression dynamics of *Rybp* and *Yaf2*. Our data showed that the *Rybp* mRNA level was gradually downregulated but that the *Yaf2* mRNA level was gradually upregulated during mESC neural differentiation (Fig. 1e). Consistently, immunoblotting analysis revealed a gradual decrease in the RYBP protein level and a gradual increase in the YAF2 protein level during mESC neural differentiation (Fig. 1f). Thus, RYBP and YAF2 have opposite expression patterns during mESC neural differentiation.

### *Rybp* loss reduces but *Yaf2* loss increases the efficiency of mESC neural differentiation

To study whether *Rybp* and *Yaf2* regulate the neural differentiation of mESCs, we generated *Rybp* knockout (*Rybp*^−/−^) and *Yaf2* knockout (*Yaf2*^−/−^) mESC clones using CRISPR/Cas9 technology (Supplementary Fig. 2c, d). *Rybp* and *Yaf2* knockouts were confirmed by DNA sequencing and Western blot/RT-qPCR analysis (YAF2 protein levels were hardly detected in mESCs) (Supplementary Fig. 2e-i). *Rybp*^−/−^ and *Yaf2*^−/−^ had no effect on the expression of RING1B, CBX7 (PRC1 subunits) or SUZ12 (PRC2 subunit) (Supplementary Fig. 3a). In addition, *Yaf2*^−/−^ had no effect on the expression of pluripotent genes, while *Rybp*^*−/−*^ slightly decreased the expression of these genes (Supplementary Fig. 3b, c), indicating that both *Rybp*^−/−^ and *Yaf2*^−/−^ have little effect on the pluripotency of mESCs. Furthermore, we could not detect any obvious change in apoptosis or the cell cycle due to these knockouts (Supplementary Fig. 3d–g).

We further induced *Rybp*^−/−^, *Yaf2*^−/−^ and wild-type mESCs to differentiate toward a neural fate. RT-qPCR analysis indicated significantly decreased expression of neuron-related genes, such as *Map2*, *Tubb3* and *Pax6*, in *Rybp*^−/−^ cells but increased expression in *Yaf2*^−/−^ cells on day 6 after neural differentiation (Fig. 1g). Furthermore, Western blot analysis indicated that *Rybp* knockout reduced, but *Yaf2* knockout increased, the protein expression of PAX6 and TUBB3 on day 6 after neural differentiation of mESCs in comparison with that in wild-type mESCs (Fig. 1h). Flow cytometry analysis also showed that *Rybp* knockout reduced the percentage of PAX6^+^ and NESTIN^+^ cells compared with wild-type mESCs on day 6 after neural differentiation (Fig. 1i, j); however, *Yaf2* knockout increased the percentage of PAX6^+^ and NESTIN^+^ cells (Fig. 1i, j).

We also performed RNA sequencing (RNA-seq) experiments with wild-type, *Rybp*^−/−^ and *Yaf2*^−/−^ neural-differentiated cells from mESCs on days 0, 3, and 6 to examine dynamic changes in gene expression upon loss of either *Rybp* or *Yaf2*. Our data revealed that *Rybp*^−/−^ resulted in significant changes in gene expression in mESCs and changed the trajectory of mESC neural differentiation (Fig. 1k and Supplementary Fig. 4a). Conversely, *Yaf2* knockout had little effect on gene expression in either mESCs or neural-differentiated cells on day 3 but led to significant changes in gene expression in neural-differentiated cells on day 6 (Fig. 1k and Supplementary Fig. 4a).

We then identified differentially expressed genes (DEGs) in *Rybp*^−/−^ and *Yaf2*^−/−^ neural differentiated cells. Consistent with the RT-qPCR results (Fig. 1g), gene ontology (GO) analysis of DEGs on day 6 after mESC neural differentiation showed that the downregulated genes in *Rybp*^−/−^ mESCs were significantly related to neural differentiation (Fig. 1l, n) and that the upregulated genes were associated with the Wnt signaling pathway (Supplementary Fig. 4b). In contrast, the genes upregulated by *Yaf2* knockout on day 6 after mESC neural differentiation were related to the central nervous system (Fig. 1m, n); the downregulated genes on days 0 and 3 were associated with negative regulation of neural development (Supplementary Fig. 4c). Overall, these results demonstrate that *Rybp*^−/−^ inhibits but *Yaf2*^−/−^ promotes the neural differentiation of mESCs, suggesting that *Rybp* and *Yaf2* play different roles in regulating the neural differentiation of mESCs.

### RYBP binds to and regulates more target genes than YAF2 in mESCs and mainly functions as a transcriptional repressor mediated by H2AK119ub

**Fig. 2:**
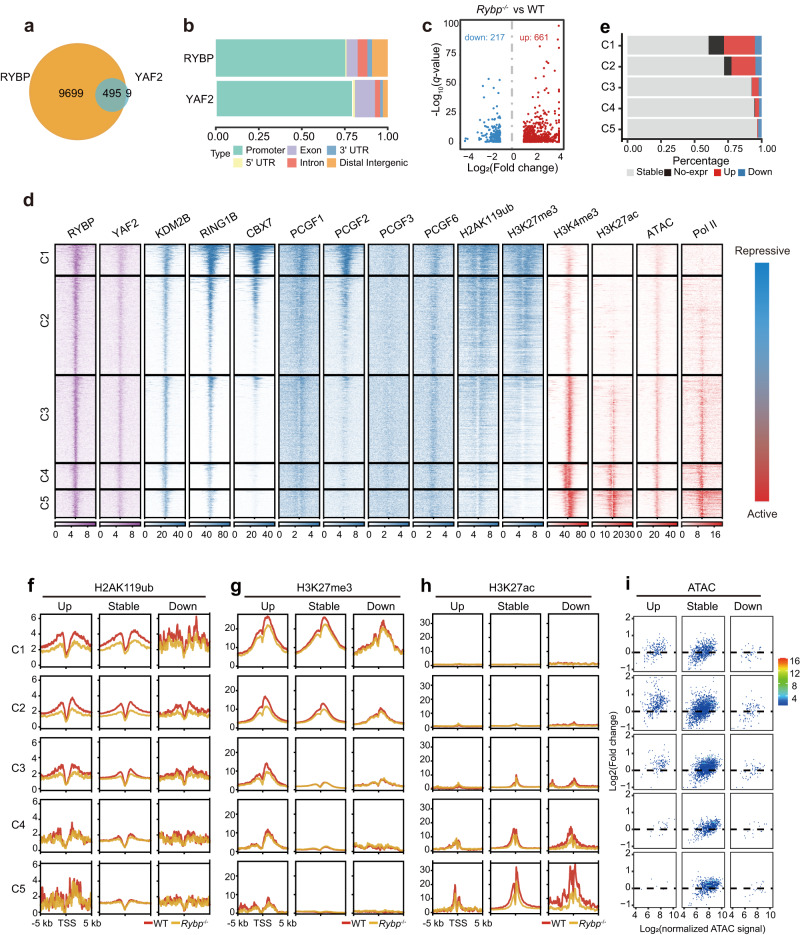
Correlation analysis of binding sites for RYBP/YAF2, other PRC1 subunits, H2AK119ub, H3K27me3, H3K4me3, H3K27ac, ATAC-seq, RNA Pol II and gene expression in wild-type and *Rybp*^*-/-*^ mESCs. **a** Venn diagram showing binding site overlap between RYBP and YAF2 in mESCs. **b** Genomic distribution of RYBP and YAF2 binding sites in mESCs. Genomic features are color-coded in the legend bar. The x-axis shows the cumulative percentage of genomic occupancy of each feature. **c** Scatter plot comparing expression changes of RYBP target genes in wild-type and *Rybp*^−/−^ mESCs. RYBP target genes were identified when RYBP bound within 1 kb of the transcription start sites (TSSs). **d** Heatmap showing enrichment of different factors (YAF2, KDM2B, RING1B, CBX7, PCGF1, PCGF2, PCGF3, PCGF6, H2AK119ub, H3K27me3, H3K4me3, H3K27ac, ATAC-seq and RNA Pol II) centered around TSSs (±5 kb) of RYBP target genes. These peaks were grouped into 5 clusters according to the enrichment of repressive and active markers. **e** Bar plots showing the proportion of different types of RYBP target genes from panel (**d**). No-expr represents genes that were defined as unexpressed. **f–h** Average enrichment of H2AK119ub (**f**), H3K27me3 (**g**), and H3K27ac (**h**) around TSS regions for upregulated, stable, or downregulated RYBP target genes from panel (**e**) between wild-type (red line) and *Rybp*^−/−^ (orange line) mESCs. **i** Scatter plots showing changes in ATAC signals at the promoters of different RYBP target genes between wild-type and *Rybp*^−/−^ mESCs.

*Rybp* expression was much higher than *Yaf2* expression in mESCs (Fig. 1e and Supplementary Fig. 2a, b), and its knockout led to abnormal expression of a large number of genes in mESCs (Supplementary Fig. 4a). Thus, we next performed chromatin immunoprecipitation followed by deep sequencing (ChIP-seq) for RYBP and YAF2 to identify their downstream targets in mESCs. A total of 10,191 and 504 binding sites were identified for RYBP and YAF2 (among them, 492 RYBP peaks overlapped with 495 YAF2 peaks), respectively, and 98.2% of YAF2 sites overlapped with RYBP sites (Fig. 2a). Gene annotation showed that RYBP and YAF2 mostly bind to gene promoter regions (Fig. 2b), indicating that RYBP and YAF2 might be directly involved in gene regulation. We further identified target genes for RYBP and YAF2, respectively. *Yaf2*^−/−^ led to significant expression changes in only a few YAF2 target genes in mESCs (Supplementary Fig. 4d), further indicating that *Yaf2* has little effect on gene expression in mESCs due to its low expression level. In contrast, *Rybp*^−/−^ resulted in differential expression of a large number of RYBP target genes in mESCs (Fig. 2c), which accounted for most of the differentially expressed genes (Supplementary Fig. 4e). *Rybp* knockout led to more than 3-fold upregulated RYBP target genes than downregulated target genes (Fig. 2c), indicating that RYBP mainly functions as a transcriptional repressor.

Variant PRC1 components colocalize not only with canonical PRC1 and PRC2^14^ but also highly with active histone markers^4^. Although RYBP could bind to 7197 gene promoters in mESCs (Fig. 2b), *Rybp* knockout affected the expression of only 878 genes (Fig. 2c). To comprehensively investigate how RYBP regulates its target genes, we collected ChIP-seq data for PRC1 components (KDM2B, RING1B, CBX7, PCGF1, PCGF2, PCGF3, PCGF6), repressive histone markers (H2AK119ub, H3K27me3), active histone markers (H3K4me3, H3K27ac), and RNA Pol II as well as ATAC-seq data (Fig. 2d). The promoter regions of RYBP target genes were clustered according to the distribution of these factors and histone modifications, and our data indicated that the repressive markers gradually weakened and the active markers gradually increased from cluster 1 (C1) to cluster 5 (C5) (Fig. 2d).

C1 and C2 share cobinding regions of cPRC1-vPRC1-PRC2-H2AK119ub-H3K27me3, which were considered repressed regions (Fig. 2d). C3, C4 and C5 contain common regions of RYBP-YAF2-KDM2B-RING1B-H3K4me3-H3K27ac-Pol II but do not contain obvious H2AK119ub and H3K27me3, which are referred to as vPRC1-only regions. The vPRC1-only regions suggested that although vPRC1 exerts a strong catalytic capacity for H2AK119ub, vPRC1 binding may not necessarily lead to the formation of obvious H2AK119ub levels at gene promoters (Fig. 2d).

By analyzing the expression of these RYBP target genes, we found that the proportion of downregulated genes in *Rybp*^*−/−*^ mESCs was almost the same in the different clusters, indicating that these genes are not directly regulated by RYBP (Fig. 2e). The proportion of derepressed genes in *Rybp*^−/−^ mESCs at the repressed regions (C1 and C2) was significantly higher than that at vPRC1-only regions (C3, C4 and C5), suggesting that H2AK119ub is more critical for gene repression than vPRC1 binding, which is consistent with the inhibitory effect of PRC1-catalyzed H2AK119ub on gene expression^30,31^. We indeed found that *Rybp* knockout significantly reduced the enrichment of H2AK119ub at the H2AK119ub-occupied RYBP target genes from C1 to C3 (Fig. 2f) but had no significant effect on RING1B enrichment (Supplementary Fig. 4f, g).

PRC1 and PRC2 tend to spatially converge on the same genomic sites to form Polycomb chromatin domains, which are uniquely enriched with H2AK119ub and H3K27me3^7,32,33^. PRC2 complexes have been divided into two mutually exclusive complexes, PRC2.1 and PRC2.2, containing either MTF2 or JARID2/AEBP2, respectively^34–36^. To investigate whether *Rybp* knockout affects genomic enrichment of EZH2 (a common PRC2 subunit), PRC2.1, PRC2.2 and H3K27me3, we performed ChIP-seq experiments using both wild-type and *Rybp*^−/−^ mESCs and found that *Rybp* knockout significantly decreased the enrichment of H3K27me3 (Fig. 2g) and weakened the recruitment of EZH2 and JARID2 to the C1, C2, and C3 regions but had little effect on MTF2 recruitment (Supplementary Fig. 4h). This is consistent with the fact that PRC2.1 can be recruited to chromatin independently but that PRC2.2 chromatin binding depends on PRC1-mediated H2AK119ub^37^.

Furthermore, bioinformatic analysis of H3K27ac ChIP-seq data indicated almost no enrichment and no change in H3K27ac in the C1, C2, and C3 regions in *Rybp*^−/−^ mESCs compared with wild-type mESCs, except for downregulation of H3K27ac in the C4 and C5 regions of downregulated genes (Fig. 2h), suggesting that H3K27ac is not required for derepression of RYBP target genes by *Rybp* loss. Moreover, compared to accessible chromatin in the regions of C4 and C5, the promoters of upregulated genes in the regions from C1 to C3 showed significantly increased chromatin accessibility in *Rybp*^−/−^ mESCs (Fig. 2i), consistent with the derepression of these genes. Together, these data indicate that RYBP mainly functions as a repressor to suppress gene expression through H2AK119ub.

### *Rybp* loss impairs neural differentiation by activating the Wnt signaling pathway and derepressing nonneuroectoderm-associated genes

**Fig. 3:**
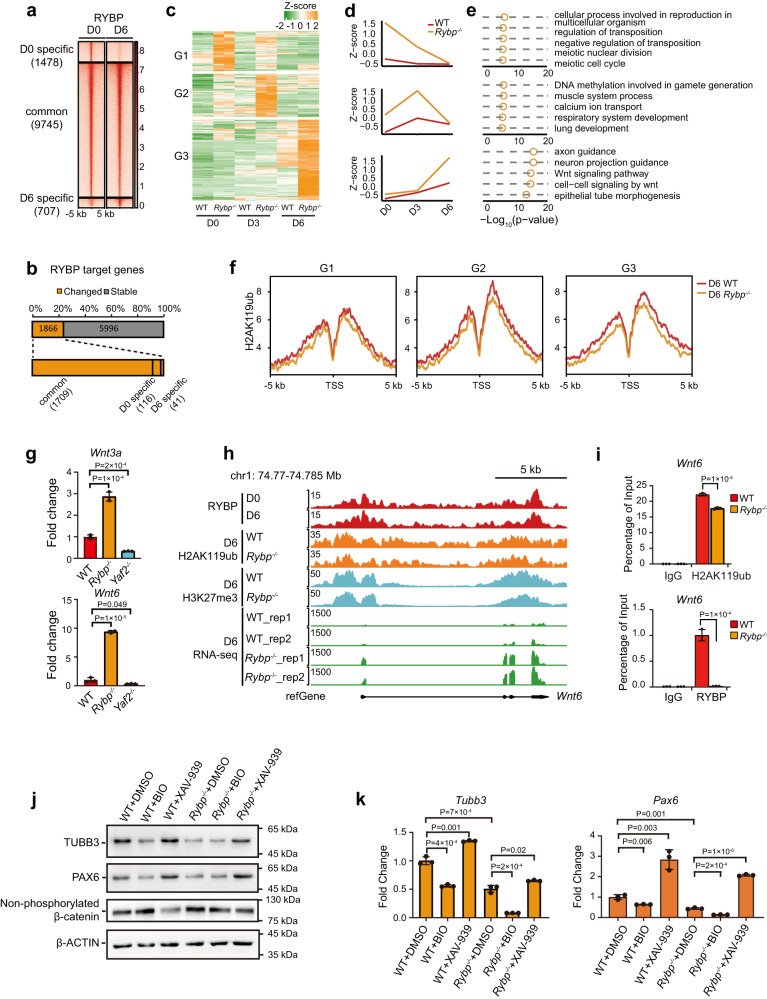
*Rybp* loss inhibits mESC neural differentiation by activating the Wnt signaling pathway and upregulating non-neuroectoderm-associated genes. **a** Heatmaps showing RYBP binding in mESCs and neural differentiated cells on day 6. The RYBP binding sites were divided into three groups based on the fold change of RYBP binding strength between day 0 and day 6. D0 specific: log_2_(D0/D6) >= 1, D6 specific, log_2_(D0/D6) <= -1. **b** Schematic diagram showing the proportion of differentially expressed or stable RYBP target genes during mESC neural differentiation in panel (**a**). **c** Clustering heatmap showing the expression pattern of upregulated RYBP target genes during neural differentiation of mESCs. **d** Line charts showing the average expression of upregulated RYBP target genes from panel (**c**) during neural differentiation of mESCs. **e** GO analysis of upregulated RYBP target genes from panel (**c**). Significance was calculated by hypergeometric distribution. **f** Average enrichment of H2AK119ub peaks in the promoters of different classes of genes from panel (**c**) between wild-type and *Rybp*^−/−^ neural-differentiated cells on day 6. **g** RT-qPCR analysis of *Wnt3a* and *Wnt6* expression in wild-type, *Rybp*^−/−^ and *Yaf2*^−/−^ neural-differentiated cells on day 6. *n* = 3 independent experiments. **h** Representative genomic tracks showing the normalized signal for RYBP, H2AK119ub, H3K27me3 and RNA-seq data at the *Wnt6* gene locus in neural-differentiated cells on day 6. **i** ChIP-qPCR showing relative enrichment of H2AK119ub and RYBP peaks at the promoter region of the *Wnt6* gene in both wild-type and *Rybp*^−/−^ neural-differentiated cells on day 6. IgG was used as the negative control. *n* = 3 independent experiments. **j** Western blot analysis of TUBB3, PAX6, and non-phosphorylated β-catenin in day 6 neural-differentiated cells from wild-type and *Rybp*^−/−^ mESCs treated with DMSO, BIO (500 ng) or XAV-939 (500 ng) from day 3 after neural differentiation. **k** RT-qPCR analysis of *Tubb3* and *Pax6* expression in the cells of panel (**j**). *n* = 3 independent experiments. For **g**, **i**, and **k**, data are represented as the mean values ± s.d.s with the indicated significance from two-sided *t* test. Source data are provided as a Source Data file.

To understand the role of RYBP during mESC neural differentiation, we further identified RYBP binding sites on day 6 in neural-differentiated cells and found that RYBP binding was redistributed compared to that on day 0 (Fig. 3a). We classified RYBP binding sites as D0- or D6-specific sites and common sites (Fig. 3a). Compared to specific sites, the binding strength of RYBP in common sites was significantly stronger (Fig. 3a). We further identified RYBP target genes during neural differentiation (on days 0, 3 and 6) by combining the RYBP binding sites on days 0 and 6 and found that 1866 RYBP target genes (1866/7862, 23.7%) were differentially expressed after *Rybp* knockout (Fig. 3b). Among these DEGs, 1709 genes (1709/1866, 91.6%) were from common sites (Fig. 3b), which showed the strongest RYBP binding. These data suggest that loss of these common RYBP binding sites in mESCs and day 6-differentiated cells has an even greater impact on gene expression.

We further extracted derepressed RYBP target genes after *Rybp* loss because derepression of these genes might be the major reason for abnormal neural differentiation. Based on clustering analysis, these genes could be classified into three groups (Fig. 3c). The genes in group 1 were mainly upregulated on day 0 (mESCs) and related to cellular reproduction and meiosis (Fig. 3d, e). The genes in group 2 were mainly upregulated on day 3 of neural differentiation and were mainly associated with muscle and respiratory system development (Fig. 3d, e). The genes in group 3 were upregulated on day 6 of neural differentiation and were associated with neural development as well as the Wnt signaling pathway (Fig. 3d, e). Enrichment of H2AK119ub at the promoters of these genes was downregulated (Fig. 3f). However, the binding of H3K27me3 showed little change after *Rybp* knockout (Supplementary Fig. 5a), which further supports that RYBP mainly regulates gene expression through H2AK119ub. We confirmed that *Rybp* knockout indeed led to significant derepression of muscle-related genes on day 3 of neural differentiation (Supplementary Fig. 5b). Wnt signaling pathway-related genes were significantly derepressed in *Rybp*^−/−^ neural differentiated cells on day 6 (Fig. 3e and Supplementary Fig. 4b), suggesting that *Rybp* knockout might slow mESC neural differentiation by activating the Wnt signaling pathway, which is consistent with its inhibitory effect on neural differentiation^38^. RT-qPCR data indicated that *Rybp* loss led to depression of *Wnt3a* and *Wnt6* (Fig. 3g), and ChIP-qPCR verified the downregulation of H2AK119ub and depletion of RYBP at the promoters of *Wnt3a* and *Wnt6* (Fig. 3h, i and Supplementary Fig. 5c, d).

To further examine whether the *Rybp*-regulated Wnt pathway is involved in neural differentiation, wild-type and *Rybp*^−/−^ mESCs were treated with BIO (Wnt agonist) and XAV-939 (Wnt inhibitor) from day 3 after mESC neural differentiation. Western blot analysis indicated that non-phosphorylated β-catenin (a marker of the active Wnt pathway) was increased in both BIO-treated cells and DMSO-treated *Rybp*^−/−^ cells but was inhibited in XAV-939-treated cells (Fig. 3j). Consistent with the finding that activation of the Wnt pathway inhibits ESC neural differentiation, Western blot and RT-qPCR analysis indicated that both BIO-treated wild-type and *Rybp*^*−/−*^ cells had reduced expression of TUBB3 and PAX6. In contrast, wild-type and *Rybp*^*−/−*^ cells treated with the Wnt inhibitor XAV-939 rescued the expression of these markers (Fig. 3j, k). In summary, these data suggest that *Rybp* loss inhibits mESC neural differentiation by activating the Wnt pathway and upregulating non-neuroectoderm-associated genes.

### *Yaf2* loss mainly leads to enhanced RYBP enrichment and inhibition of YAF2-RYBP cotargeted genes through RYBP-mediated H2AK119ub

**Fig. 4:**
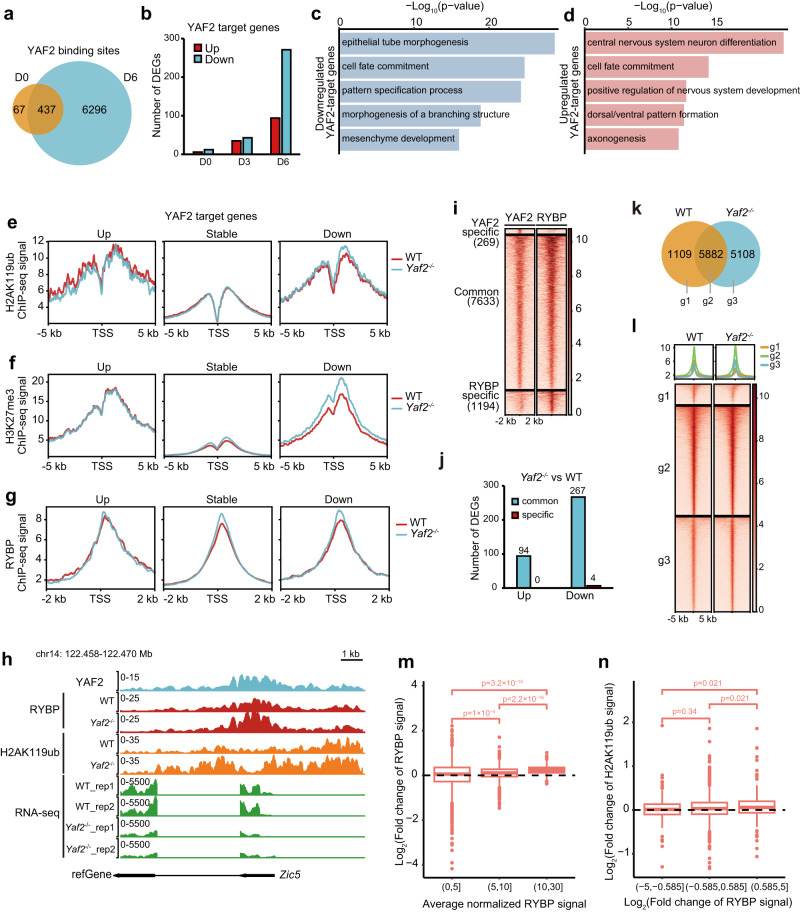
*Yaf2* loss mainly leads to inhibition of YAF2-RYBP co-targeted genes through RYBP-mediated H2AK119ub. **a** Venn diagram showing the overlap of YAF2 binding sites in mESCs and neural differentiated cells on day 6. **b** Bar plots showing the number of YAF2 target DEGs in mESCs and neural differentiated cells on days 0, 3, and 6, respectively. **c**, **d** GO analysis of downregulated (**c**) and upregulated (**d**) YAF2 target genes after *Yaf2* knockout in neural-differentiated cells on day 6. **e**–**g** Average normalized ChIP signal for H2AK119ub (**e**), H3K27me3 (**f**) and RYBP (**g**) in the promoters of upregulated, stable, and downregulated YAF2 target genes after *Yaf2* knockout. **h** Genomic tracks showing the enrichments of YAF2/RYBP/H2AK119ub and mRNA level at the *Zic5* gene locus in day 6 neural-differentiated cells. **i** Heatmaps showing YAF2 and RYBP binding in day 6 neural-differentiated cells. YAF2 specific, log_2_(YAF2/RYBP) >= 1; RYBP specific, log_2_(YAF2/RYBP) <= −1. **j** Bar plots showing the number of YAF2-target DEGs derived from the common and YAF2-specific regions in panel (**i**). **k** Venn plot showing RYBP peak overlap between wild-type and *Yaf2*^−/−^ neural differentiated cells on day 6. **l** Heatmaps and profiles showing normalized RYBP ChIP signals between wild-type and *Yaf2* neural differentiated cells on day 6 based on (**k**). **m** Box plots showing the distribution of Log_2_(fold change) of RYBP peaks between wild-type and *Yaf2*^−/−^ neural-differentiated cells on day 6. The RYBP binding sites (*n* = 4035) were divided into three groups based on the average peak strength. **n** Box plots showing the distribution of Log_2_(fold change) of H2AK119ub peaks between wild-type and *Yaf2*^−/−^ neural-differentiated cells on day 6. H2AK119ub sites (*n* = 4035) were divided into three groups based on the change of RYBP peaks between wild-type and *Yaf2*^−/−^ neural-differentiated cells on day 6. The upper and lower edges of the boxes represent 75% and 25% quartiles, the central line represents the median, the whiskers extend to 1.5 × IQR, and the dots represent outliers in **m** and **n**. Significant results for **m** and **n** were derived from the two-sided Wilcoxon test. Source data are provided as a Source Data file.

Both YAF2 and RYBP can promote RING1B-dependent H2AK119 ubiquitination^22^. However, *Yaf2* knockout promoted mESC neural differentiation, whereas *Rybp* knockout inhibited mESC neural differentiation (Fig. 1). To investigate the mechanisms of *Yaf2* knockout in promoting mESC neural differentiation, we performed YAF2 ChIP-seq experiments in neural-differentiated cells and identified YAF2 binding sites on day 6 with high quality. Compared to the peaks identified on day 0, the number of YAF2 binding sites increased significantly on day 6, and the majority of YAF2 binding sites on day 0 overlapped with those on day 6 (Fig. 4a). Integration analysis between YAF2 ChIP-seq data and RNA-seq data indicated that only a few YAF2 target genes were differentially expressed in *Yaf2*^−/−^ mESCs and *Yaf2*^−/−^ neural differentiated cells on day 3 in contrast to wild-type cells, although a large number of YAF2 target genes were differentially expressed on day 6 of mESC neural differentiation (Fig. 4b), suggesting that YAF2 plays critical roles in regulating gene expression on day 6 of mESC neural differentiation. Therefore, we mainly focused on YAF2 target DEGs on day 6.

The DEG results showed that *Yaf2* loss mainly resulted in the downregulation of its target genes in neural-differentiated cells on day 6 (Fig. 4b). GO analysis showed that the downregulated genes were associated with non-neuroectoderm differentiation (Fig. 4c), while the upregulated genes were associated with neural differentiation (Fig. 4d), indicating that *Yaf2* loss led to significant derepression of neural-related genes and inhibition of non-neuroectoderm-associated genes, which was consistent with the promoting effect on neural differentiation by *Yaf2* knockout.

We further investigated the regulatory mechanism of YAF2 on its target genes. YAF2 belongs to the non-classical PRC1 subunit and has the ability to enhance H2AK119ub deposition by promoting PRC1 activity^22^. We performed ChIP-seq for RING1B, RYBP, H2AK119ub, and H3K27me3 and analyzed their enrichment at the promoters of YAF2 target genes. Our results showed that *Yaf2* knockout had no significant effect on RING1B binding in either mESCs or neural-differentiated cells on day 6 (Supplementary Fig. 5e, f). Surprisingly, the enrichment of both H2AK119ub and H3K27me3 was enhanced at the promoters of downregulated genes but remained unchanged at the promoters of upregulated and stable genes after *Yaf2* knockout (Fig. 4e, f). Similarly, the enrichment of RYBP at the promoters of upregulated genes was not affected but increased at the promoters of downregulated genes (Fig. 4g), which was exemplified at the *Zic5* gene locus (Fig. 4h). Based on these results, it seems that YAF2 might have no direct role in derepressed target genes after the loss of *Yaf2*. Considering that YAF2 competes with RYBP binding, its loss might regulate gene expression by influencing RYBP binding and H2AK119ub deposition.

To explore how RYBP binding is affected by *Yaf2* knockout, we first examined RYBP expression at both the mRNA and protein levels and found that RYBP expression slightly increased in *Yaf2*^−/−^ neural-differentiated cells on day 6 compared with wild-type cells (Fig. 1h and Supplementary Fig. 5g). Next, we compared YAF2 binding sites with RYBP in wild-type neural-differentiated cells on day 6 and found that most of the YAF2 binding sites were colocalized with RYBP (Fig. 4i), and approximately 99% (94/94 upregulated genes, 267/271 downregulated genes) of the *Yaf2*^*−/−*^ differential target genes came from the colocalization regions (Fig. 4j), supporting that *Yaf2* loss regulated gene expression by affecting RYBP binding. Then, we investigated the effect of *Yaf2* knockout on RYBP distribution and observed that *Yaf2* loss did not lead to RYBP redistribution to new sites but resulted in the redistribution of its binding strength (Fig. 4k, l). We further investigated the features of RYBP redistribution and found that the regions with strong RYBP binding tended to become even stronger, and the weak binding sites with RYBP tended to become even weaker after *Yaf2* loss (Fig. 4m). Consistently, we also found an increase in YAF2 binding upon *Rybp* loss at the selected RYBP sites with strong binding signals in ESCs (Supplementary Fig. 5h). These findings were in line with our discovery of YAF2/RYBP competitive binding (Fig. 1). To further explore whether redistribution of RYBP binding strength influences H2AK119ub, we divided RYBP binding sites into three groups based on changes in RYBP binding after *Yaf2* knockout and found that the regions with enhanced RYBP binding showed an elevation in H2AK119ub deposition (Fig. 4n).

*Rybp* loss derepressed Wnt pathway-related genes, while *Yaf2* loss led to slight inhibition of Wnt-related genes (Fig. 3g). However, the enrichment of RYBP and H2AK119ub was not significantly affected in the promoters of *Wnt3a*, *Wnt6a* and *Wnt9* genes by loss of *Yaf2* (Supplementary Fig. 5i), suggesting that YAF2 might indirectly regulate Wnt-related genes. Taken together, our results showed that *Yaf2* loss resulted in the redistribution of RYBP binding strength and caused the higher enrichment of RYBP at the RYBP-YAF2 cobound sites, which might enhance H2AK119ub deposition and further depress the non-neuroectoderm-associated genes.

## Discussion

**Fig. 5:**
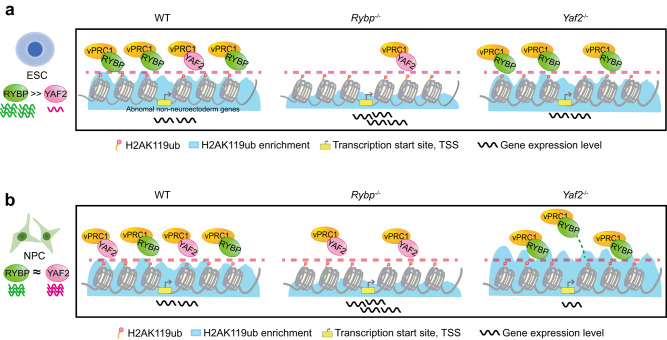
Model of RYBP-vPRC1 and YAF2-vPRC1 for H2AK119ub enrichment and gene regulation during mESC neural differentiation. **a** In mESCs, *Rybp* is highly expressed compared with *Yaf2*. *Rybp* knockout leads to a significant reduction in H2AK119ub enrichment and a large number of genes that are derepressed; *Yaf2* knockout has little effect on H2AK119ub enrichment and gene expression. **b** *Rybp* gradually decreases but *Yaf2* gradually increases during mESC neural differentiation. RYBP and YAF2 interact with vPRC1 in a competitive manner. *Rybp* knockout increases the occupation of YAF2 in RYBP-YAF2 cobinding regions but decreases H2AK119ub enrichment and leads to derepression of these cobinding genes. In contrast, *Yaf2* knockout increases the occupation of both RYBP and H2AK119ub on RYBP-YAF2 cobinding regions and downregulates the expression of these cobinding genes.

RYBP and its paralog YAF2 are often mentioned together due to their high protein sequence similarity. RYBP and YAF2 compete for RING1A/B with CBXs, and both can dramatically stimulate the activity of RING1B toward H2AK119ub^13,22,39^. Biochemical approaches have revealed that both RYBP and YAF2 stimulate similar levels of PCGF1-RING1B E3 ligase activity in vitro^22^, but it is difficult to quantitatively measure this ability of RYBP or YAF2 to stimulate RING1B E3 ligase activity in vivo due to the complexity of the intracellular environment. Whether RYBP and YAF2 have different biological functions is still unclear. In this study, we found that RYBP and YAF2 are not redundant during mESC neural differentiation, and *Rybp* loss decreased while *Yaf2* loss increased the efficiency of mESC neural differentiation (Fig. 5). An earlier study also suggested that RYBP and YAF2 might have different functions in regulating gene expression^25^.

RYBP and YAF2 compete to bind RING1B, which was consistent with a previous report that RYBP and YAF2 mutually exclusive bind to the same RING1B surface^40^, and our results showed that they share similar binding sites in both mESCs and NPCs in the genome, and loss of either RYBP or YAF2 resulted in the redistribution of the other. These results were similar to those of paralogous transcription factors competing to bind the genome^41^, and our previous work also showed that CTCF and its short isoform CTCF-s competitively bind to the genome but share the majority of binding sites^42^, further supporting our findings in this work.

We found that H2AK119ub catalyzed by vPRC1 was critical for gene repression, but *Rybp* knockout, which dramatically decreased H2AK119ub, did not derepress all its target genes. We believe that gene derepression requires not only the removal of inhibitory marks such as H2AK119ub and H3K27me3 but also activation factors such as transcription factors, active histone marks, and accessible chromatin. For example, RYBP and H2AK119ub were enriched in the promoters of Wnt pathway genes (*Wnt3a* and *Wnt9a*) in both mESCs and neural differentiated cells on day 6. Consistently, their expression was extremely low in mESCs, and even *Rybp* knockout did not lead to their significant upregulation. However, they were highly expressed in neural differentiated cells on day 6, indicating the existence of active factors, and their expression was dramatically upregulated after *Rybp* knockout. These results indicate that the changes in gene expression are balanced between active and inhibitory factors.

Taken together, our findings provide the insight that the diversity of PRC1 subunits RYBP and YAF2 contributes to meticulous and accurate gene expression during mESC neural differentiation.

## Methods

### Cell culture and differentiation

46C Sox1-GFP mES cell lines^4^ were cultured on 0.2% gelatin-coated plates in Dulbecco’s Modified Eagle Medium (DMEM) high-glucose medium (HyClone) containing 15% fetal bovine serum (FBS, Gibco), 1% GlutaMAX (Gibco), 0.1 mM 2-mercaptoethanol (Gibco), 1% non-essential amino acids (Gibco), 1% sodium pyruvate (Gibco), 1000 U/ml leukemia inhibitory factor (LIF) and 2i inhibitors (3 μM CHIR99021 and 1 μM PD0325901). HEK293T cells were maintained in DMEM high-glucose medium supplemented with 10% FBS (Natocor). All cell lines were cultured under 5% CO_2_ at 37 °C.

mESCs were induced to undergo neural differentiation as previously described^43^. Cells were dissociated and plated at a density of 1 × 10^4^ cells/cm^2^ on Attachment Factor (Gibco, S006100)-coated plates in DMEM/F12 (1:1) medium supplemented with 0.5% N2 (Gibco), 1% B27 (Gibco), 1% non-essential amino acids, 1% Glutamax, and 0.1 mM 2-mercaptoethanol (Gibco). Fresh culture medium was changed every other day.

### Plasmid construction

The plasmids for protein purification of the pull-down assay were cloned and inserted into the pGEX-4T or pET-28a vector (between EcoR1 and Xhol1), which contains sequences encoding GST-tag or 6×His-tag at the N-terminus. The coding region of *Ring1b* or *Yaf2* was amplified from mouse cDNAs by PCR. The plasmids for Flag-IP were cloned and inserted into the pSIN vector (between EcoR1 and Cla1). The coding regions of *Rybp*, *Yaf2*, *Ring1b* and their deletions were amplified from mouse cDNAs by PCR. All the constructs were confirmed by Sanger sequencing. The plasmids and primers used in this study are shown in Supplementary Table S1.

### CRISPR/Cas9 genome editing

*Rybp*^−/−^ and *Yaf2*^−/−^ mESC lines were generated by using the CRISPR/Cas9 method as previously described^44^. In brief, sgRNAs targeting *Yaf2* and *Rybp* in the mouse genome were designed by using https://portals.broadinstitute.org/gpp/public/analysis-tools/sgrna-design. The primers used to construct individual sgRNAs are shown in Supplementary Table S2. The pX330 plasmid (Addgene plasmid #42230), which contains sgRNA, and the pMD-18T plasmid, which contains donor repair templates, were electroporated into 46C mESCs. The cells were selected with 2 μg/ml puromycin (Gibco) for at least four days. Individual clones were replated on gelatin-coated 48-well plates for further selection. The clones were expanded, and the target loci were sequenced by Sanger sequencing.

### Immunoprecipitation and Western blot

Cells were collected and kept at -80 °C until immunoprecipitation (IP). Cells were resuspended in cold lysis buffer (10 mM Tris-HCl (pH 8.0), 10 mM NaCl, 3 mM MgCl_2_, 0.5% IGEPAL-CA630, 1 mM EDTA and 1 × protease inhibitor cocktail (PIC, Bimake, B14001)) on ice for 30 min with occasional pipetting up and down and centrifuged at 12,000 × *g* at 4 °C for 5 min to remove insoluble material. The protein concentration of the supernatant was measured by nanodrop and diluted with IP buffer (20 mM Tris-HCl (pH 8.0), 150 mM NaCl, 10% glycerol, 0.5% Triton X-100, 1 mM EDTA and 1 × PIC) and then incubated with specific antibodies and Dynabeads protein A/G (1:1 mixed) at 4 °C for 4 hrs. The beads were washed three times with IP Wash buffer (20 mM Tris-HCl (pH 8.0), 150 mM NaCl, 0.5% Triton X-100 and 1 mM EDTA), and then protein bound on the beads was boiled with 2 × SDS loading buffer for 10 min. The eluted bound proteins were analyzed by Western blotting.

For Western blot experiments, cells were resuspended and sonicated in RIPA buffer (50 mM Tris-HCl (pH 7.4), 150 mM NaCl, 1 mM EDTA, 1% Triton X-100, 0.1% sodium deoxycholate, 0.1% SDS and 1 × PIC). After centrifugation at 12,000 × *g* at 4 °C for 10 min, soluble proteins were quantified. After SDS-polyacrylamide gel electrophoresis (PAGE), proteins were transferred onto polyvinylidene fluoride membranes (PVDF). The PVDF membrane was blocked with 5% milk in TBS-T (TBS with 0.05% Tween-20). The PVDF membrane was incubated with the corresponding primary antibody and secondary antibody. The antibodies used in this study are listed in Supplementary Table S3.

### Pull-down assays

GST-RING1B and His-mYAF2-mCherry fusion proteins were expressed in *E. coli* BL21 (DE3) (TransGen Biotech, CD601), purified and quantified using the Pierce BCA Protein Assay Kit (Thermo Scientific, 23225). Three hundred nanograms of GST-RING1B protein was incubated with 1 mg of total cell extracts of ESCs as well as His-mYAF2-mCherry protein at increasing concentrations (0, 100, and 300 ng) in PBS buffer supplemented with 1 × PIC at 4 °C for 4 hrs. GSTSep Glutathione Agarose Resin was added to the mixture and incubated at 4 °C for 2 hrs. Then, the resin was washed four times with PBS buffer and boiled in SDS loading buffer for Western blotting.

### Quantitative RT-PCR

The cells were collected and washed with PBS, and the total RNA was extracted with a RaPure Total RNA Micro Kit (Magen, 4012-03). For quantitative PCR, cDNAs were synthesized with HiScript® III RT SuperMix for qPCR (Vazyme Biotech, R323-01). Real-time PCR was performed using SYBR Green mix (Genstar, A301-01) on a CFX96 real-time PCR system (Bio-Rad) according to the manufacturer’s instructions. The data were analyzed by using the ΔΔC_t_ method^45^, and *Gapdh* was used as an internal control. All experiments were repeated three times. The primers used for the RT-qPCR assays are listed in Supplementary Table S4.

### Protein expression and purification

pET-28a-m*Yaf2*-mCherry plasmids were expressed in *E. coli* BL21 (DE3), and transformed cells were grown at 37 °C with kanamycin to a density of 0.6 to 0.8 at OD600 (optical density at 600 nm) and induced with 0.5 mM isopropyl-β-D-thiogalactopyranoside (IPTG) at 25 °C for 5 hrs. The cells were collected and resuspended in lysis buffer (25 mM Tris-HCl (pH 7.5), 150 mM NaCl, 5% glycerol, 1 × PIC and 20 mM imidazole) and crushed by using a low-temperature ultra-high pressure continuous flow cell crusher (JNBIO, LC-10C). Lysates were cleared by centrifugation at 12,000 × g at 4 °C for 45 min, and protein supernatants were allowed to flow through a Ni-NTA Sefinose Column (Sangon Biotech, C600791). The columns were washed three times with lysis buffer containing 20 mM imidazole and then washed twice with lysis buffer containing 30 mM imidazole. The proteins were eluted from the Ni-NTA column with elution buffer (25 mM Tris-HCl (pH 7.5), 500 mM NaCl, 10% glycerol, 1 × PIC, and 300 mM imidazole). The eluted fraction was dialyzed in high salt buffer (25 mM Tris-HCl (pH 7.5), 500 mM NaCl, 10% glycerol, and 1 mM DTT) at 4 °C to remove imidazole. The proteins were concentrated with Amicon Ultra Centrifugal filters (Millipore, 30 K MWCO), and the protein concentration was determined by using the Pierce BCA Protein Assay Kit (Thermo Scientific, 23225).

pGEX-4T-m*Ring1b* was transformed and expressed in *E. coli* BL21 (DE3). The experimental procedures for GST-fusion protein purification were the same as those mentioned above. After centrifugation, the supernatant was collected and mixed with GSTSep Glutathione Agarose Resin (Yeasen, 20507ES10) equilibrated with PBS buffer. Then, the mixtures of protein and GST Agarose Resin were rotated at 4 °C for 2 hrs, washed four times with T buffer (50 mM Tris-HCl (pH 7.5), 1 mM DTT, 0.1% NP-40, and 10% glycerol) containing 150 mM NaCl and eluted with T buffer by adding 25 mM glutathione. The eluted fraction was dialyzed in T buffer containing 300 mM NaCl, concentrated, and stored at -80 °C.

### RNA-seq

The total RNAs were extracted with a RaPure Total RNA Micro Kit (Magen, R4012-03). RNA sequencing libraries were constructed using a VAHTS Universal V8 RNA-seq Library Prep Kit for Illumina (Vazyme Biotech, NR605) according to the manufacturer’s instructions. In brief, 500 ng of total RNAs were used for RNA-seq library construction. Poly(A)-containing mRNAs were isolated by poly(A) selection beads (Vazyme Biotech, N401) and further reverse transcribed to cDNAs. cDNAs were ligated with adapters, amplified by PCR, and purified with AMPure XP beads (Beckman Coulter, A63882) to obtain the final sequencing library. The libraries were sequenced on an Illumina NovaSeq instrument.

### RNA-seq data analysis

The raw paired-end reads were trimmed using Trim_Galore (v0.6.5) and then mapped and quantified to the mouse mm10 genome with the STAR-RSEM pipeline using RSEM (v1.2.22)^46^. Transcript-level counts were collapsed to gene-level counts using tximport (v1.20.0)^47^, and differential gene expression was analyzed with DESeq2 (v1.32.0)^48^. Genes with a fold change larger than 2 and a q-value less than 0.05 were considered differentially expressed genes (DEGs). GO analysis was conducted with clusterProfiler (v4.0.0)^49^. Genes with transcripts per million (TPM) values generated by RSEM software less than 0.5 in both duplicates were considered unexpressed.

### Chromatin immunoprecipitation

For RYBP, YAF2, MTF2, JARID2, EZH2 and RING1B, ChIP was performed as previously described^50^. In brief, 10^7^ cells were crosslinked with 1% formaldehyde at room temperature (RT) for 10 min, and the reaction was stopped by adding 0.125 M glycine. Crosslinked cells were lysed in ChIP SDS lysis buffer (1% SDS, 10 mM EDTA, 50 mM Tris-HCl (pH 8.0) and 1 × PIC) and then sonicated to achieve chromatin fragments sized 200-400 bp. The chromatin was diluted 9-fold with dilution buffer (0.01% SDS, 1.1% Triton X-100, 1.2 mM EDTA, 16.7 mM Tris-HCl (pH 8.0) and 167 mM NaCl). The supernatant was precleared with Dynabeads protein A/G (1:1 mixed) for 1 hr at 4 °C and then immunoprecipitated using 5 μg antibodies for 12 hrs and 50 μl Dynabeads protein A/G (1:1 mixed) for 2 hrs. Immune complexes were washed once with the following buffers: low salt wash buffer (0.1% SDS, 1% Triton X-100, 2 mM EDTA, 20 mM Tris-HCl (pH 8.0) and 150 mM NaCl), high salt wash buffer (0.1% SDS, 1% Triton X-100, 2 mM EDTA, 20 mM Tris-HCl (pH 8.0), and 500 mM NaCl), LiCl wash buffer (250 mM LiCl, 1% IGEPAL-CA630, 1% deoxycholic acid (sodium salt), 1 mM EDTA and 10 mM Tris-HCl (pH 8.0)) and twice with TE buffer (10 mM Tris-HCl (pH 8.0) and 1 mM EDTA).

For native calibrated ChIP-seq experiments of H2AK119ub and H3K27me3, 5 × 10^6^ mESCs or differentiated neural progenitor cells (NPCs) were collected and resuspended in ice-cold lysis buffer (10 mM Tris-HCl (pH 8.0), 10 mM NaCl, 3 mM MgCl_2_, 0.5% IGEPAL-CA630, 1 × PIC). Nuclei were then washed and resuspended in 100 μl of MNase digestion buffer (10 mM Tris-HCl (pH 8.0), 10 mM NaCl, 3 mM MgCl_2_, 3 mM CaCl_2_, 0.5% IGEPAL-CA630 and 1 × PIC). Each sample was incubated with 250 gel units of MNase (NEB, M0247S) at 37 °C for 7 min, followed by the addition of 4 mM EDTA to halt MNase digestion. After centrifugation at 1500 × *g* at 4 °C for 5 min, the supernatant was retained at 4 °C and named S1. The pellet was incubated with 50 μl nucleosome release buffer (10 mM Tris-HCl (pH 7.5), 10 mM NaCl, 0.2 mM EDTA and 1 × PIC) at 4 °C for 1 hr, with occasional pipetting up and down, and then passed twenty times through a 27 G needle. Following centrifugation at 1500 × g for 5 min at 4 °C, supernatant 2 (named S2) was collected and combined with S1. Forty nanograms of spike-in chromatin (Active Motif, 53083) was added to 25 μg of S1 + S2 chromatin for calibration. For ChIP experiments, S1/S2 nucleosomes were diluted 9-fold in native ChIP incubation buffer (70 mM NaCl, 10 mM Tris (pH 7.5), 2 mM MgCl_2_, 2 mM EDTA, 0.1% Triton X-100 and 1 × PIC). The supernatant was precleared with Dynabeads protein A/G (1:1 mixed) at 4 °C for 1 hr and then immunoprecipitated using 3 μg antibodies for 12 hrs and 30 μl Dynabeads protein A/G (1:1 mixed) for 2 hrs. Immune complexes were washed twice with Native ChIP wash buffer I (20 mM Tris (pH 7.5), 2 mM EDTA, 125 mM NaCl and 0.1% Triton X-100), once with Native ChIP wash buffer II (20 mM Tris (pH 7.5), 2 mM EDTA, 250 mM NaCl and 0.1% Triton X-100) and once with TE buffer (10 mM Tris-HCl (pH 8.0) and 1 mM EDTA).

ChIPed DNA was eluted with 200 μl of freshly prepared elution buffer (1% SDS, 0.1 M NaHCO_3_) with 125 mM NaCl and 400 μg/ml Proteinase K at 65 °C for 8 hrs for reverse-crosslinking and purified with a MinElute PCR Purification Kit (QIAGEN, 28106) for ChIP-qPCR or ChIP-seq. ChIP-seq libraries were constructed using the VAHTS Universal V3 DNA Library Prep Kit for Illumina (Vazyme, ND 607) according to the manufacturer’s instructions. The primers used for ChIP-qPCR are listed in Supplementary Table S5.

### ChIP-seq data analysis

Adaptors and low-quality reads were trimmed with Trim_Galore (v0.6.5). For RYBP, YAF2, RING1B, and H3K27ac ChIP-seq, the trimmed reads were aligned to the mouse mm10 genome using Bowtie2 (v2.2.5)^51^ with the parameter “--very-sensitive --end-to-end --no-unal”. For H2AK119ub and H3K27me3 ChIP-seq, the trimmed reads were mapped to mm10 (mouse) and dm6 (drosophila melanogaster) reference genomes using Bowtie2 (v2.2.5) with the parameters “--very-sensitive --end-to-end --no-unal --no-mixed --no-discordant”. For EZH2, JARID2, and MTF2 ChIP-seq, the trimmed reads were mapped to mm10 (mouse) and hg38 (human) reference genomes using Bowtie2 (v2.2.5) with the same parameters as above. The mapped reads with a quality lower than 30 were filtered out. Duplicated reads were removed with sambamba (v0.6.7). The reads overlapping with mouse mm10 blacklist regions (http://mitra.stanford.edu/kundaje/akundaje/release/blacklists) were excluded.

For RING1B ChIP-seq, uniquely mapped reads for each sample were subsampled to 10 million, and replicates were subjected to DiffBind (v3.2.4)^52^ for differential binding analysis. For calibrated ChIP-seq (cChIP-seq), the reads mapped to the mm10, dm6, or hg38 genome were extracted and calculated for calibration as previously described^53^. BamCoverage (v3.5.1) from deepTools^54^ was used to generate the normalized bigwig files with the parameter “--normalizeUsing RPGC” for normal ChIP-seq and “--normalizeUsing RPGC --scaleFactor” for cChIP-seq. The reads mapped to the mm10 genome were used to identify peaks by MACS2 (v2.2.7.1)^55^ in broad mode with the default parameters, except with the parameter “-q 0.05” for RYBP and YAF2. Peak annotation was performed with ChIPseeker (v1.28.3)^56^. Target genes of both RYBP and YAF2 were identified when they bound within 1 kb of the transcription start sites (TSSs). Mapping statistics and peak calling information for ChIP-seq data are listed in Supplementary Table S6, and the normalization information for cChIP-seq data is listed in Supplementary Table S7.

### ATAC-seq

ATAC-seq was performed as described previously^50^. In brief, 5×10^4^ cells were collected and washed once with 50 μl of cold PBS. Then, the cells were resuspended in 50 μl of lysis buffer (10 mM Tris-HCl (pH 7.4), 10 mM NaCl, 3 mM MgCl_2,_ and 0.2% (v/v) IGEPAL CA-630). Then, the suspension of nuclei was centrifuged at 500 × *g* at 4 °C for 10 min. The pellet was further resuspended by adding 50 μl transposition reaction mix (10 μl TD buffer, 5 μl Tn5 transposase, and 35 μl nuclease-free H_2_O) (Vazyme Biotech, TD501-01) and incubated at 37 °C for 30 min. DNA was isolated using a MinElute PCR Purification Kit (QIAGEN, 28106). ATAC-seq libraries were generated using the TruePrep DNA Library Prep Kit V2 for Illumina (Vazyme Biotech, TD501-01) and purified with AMPure XP beads (Beckman).

### ATAC-seq data analysis

Paired-end reads were treated with trim_galore (v0.6.5) to remove adapters and low-quality reads and then aligned to the mm10 genome by using Bowtie2 (v2.2.5) with the parameters “--very-sensitive --end-to-end --no-unal -X 2000”. The alignment files were further processed with sambamba (v0.6.7) to remove low-quality mapped reads and duplicates. The reads overlapping with mouse mm10 blacklist regions (http://mitra.stanford.edu/kundaje/akundaje/release/blacklists) were filtered out. Accessible regions were identified using MACS2 (v2.2.7.1) without control using the default parameters, and differential analysis was performed using DiffBind (v3.2.4). Normalized bigWig files were generated using bamCoverage (v3.5.1). Mapping statistics and peak calling information for ATAC-seq data are listed in Supplementary Table S8.

### Flow cytometry

Apoptosis assay and cell cycle analysis were performed as described previously^57^. mESCs were dissociated by 0.25% Trypsin-EDTA (except apoptosis assay without EDTA) and quenched with serum-containing medium. Day 6 differentiated cells were collected with Accutase (STEM CELL, 7920). Then, the cells were prepared for apoptosis by using the Apoptosis Detection Kit (Vazyme Biotech A211-02) and analyzed for cell cycle detection with the Cell Cycle Detection Kit (KeyGen, KGA512). To evaluate the neural differentiation efficiency, the cells were fixed with 4% paraformaldehyde at RT for 15 min, permeabilized with 0.3% Triton X-100 at RT for 15 min, and blocked with 3% bovine serum albumin (BSA) at RT for 1 hr. Then, the cells were stained with a mixture of anti-rabbit PAX6 antibody (Biolegend, 901301, dilution 1:100) and anti-mouse NESTIN antibody (CST, 33475, dilution 1:100) at RT for 1 hr. After washing with cold PBS twice, the cells were stained with a mixture of goat anti-mouse IgG Alexa 594 secondary antibody (Invitrogen, Cat# A11032, dilution 1:500) and goat anti-rabbit IgG Alexa 647 secondary antibody (Invitrogen, Cat#21245, dilution 1:500) at RT for 1 hr. Then, the cells were washed with cold PBS twice, resuspended in single-cell suspension in FACS buffer (DPBS containing 5% FBS) and passed through a cell strainer (70 μm). Afterward, the cells were analyzed using LSR Fortessa SORP (BD Biosciences), and the data were analyzed using FlowJo (v10). Standard forward and side scatter gating was used to exclude debris and isolate single cells for further analysis. The unstained cells were gated as the negative control, and PAX6-stained cells or NESTIN-stained cells were used as the positive control.

### Statistics and reproducibility

The number of biological and technical replicates are reported in the legend of each figure. All statistical analysis methods are indicated in the figure legends and Methods. The data are presented as the mean value ± s.d.s unless otherwise indicated in the figure legend. Statistical significance in the two-group comparisons was determined by Student’s *t*-test (two-tailed). The p values are indicated in the figure legends. All data from representative experiments were repeated at least two times independently with similar results obtained.

### Reporting summary

Further information on research design is available in the Nature Portfolio Reporting Summary linked to this article.

### Supplementary information

Supplementary Information

Reporting Summary

### Source data

Source data

## Data Availability

The ATAC-seq, ChIP-seq and RNA-seq data reported in this paper have been deposited in the Genome Sequence Archive database^58^ in the National Genomics Data Center^59^ (GSA: CRA006987) and in the Gene Expression Omnibus (GEO) database (GSE213416). The dataset of this paper has been submitted to the figshare repository (10.6084/m9.figshare.24407761.v1). Access codes for the published data used in Fig. 2d are as follows: GSE40860 for KDM2B and RING1B; GSE122715 for PCGF1, PCGF2, PCGF3, PCGF6, H3K27me3 and H3K4me3; GSE76823 for H2AK119ub; and GSE64825 for Pol II. Any additional information required to reanalyze the data reported in this paper is available from the lead contact upon request. Source data are provided with this paper.
