# Supplementary material for: Functional dissection of PRC1 subunits RYBP and YAF2 during neural differentiation of embryonic stem cells
Source: Nat Commun. 2023 Nov 7;14:7164. doi: 10.1038/s41467-023-42507-9 (PMC10630410; doi:10.1038/s41467-023-42507-9)
Supplement: Supplementary file 1 — Supplementary Information [file 41467_2023_42507_MOESM1_ESM.pdf]

Supplementary Information for

Functional dissection of PRC1 subunits RYBP and YAF2 during neural differentiation of embryonic stem cells

Yanjiang Liu†, Gongcheng Hu†, Shengxiong Yang, Mingze Yao, Zicong Liu, Chenghong Yan, Yulin Wen, Wangfang Ping, Juehan Wang, Yawei Song, Xiaotao Dong, Guangjin Pan, Hongjie Yao*

† Contributed equally to this work.

* Corresponding author.

This PDF file includes:

Supplementary Figures 1 to 5;

Supplementary Tables 1 to 8.

Supplementary Figure 1

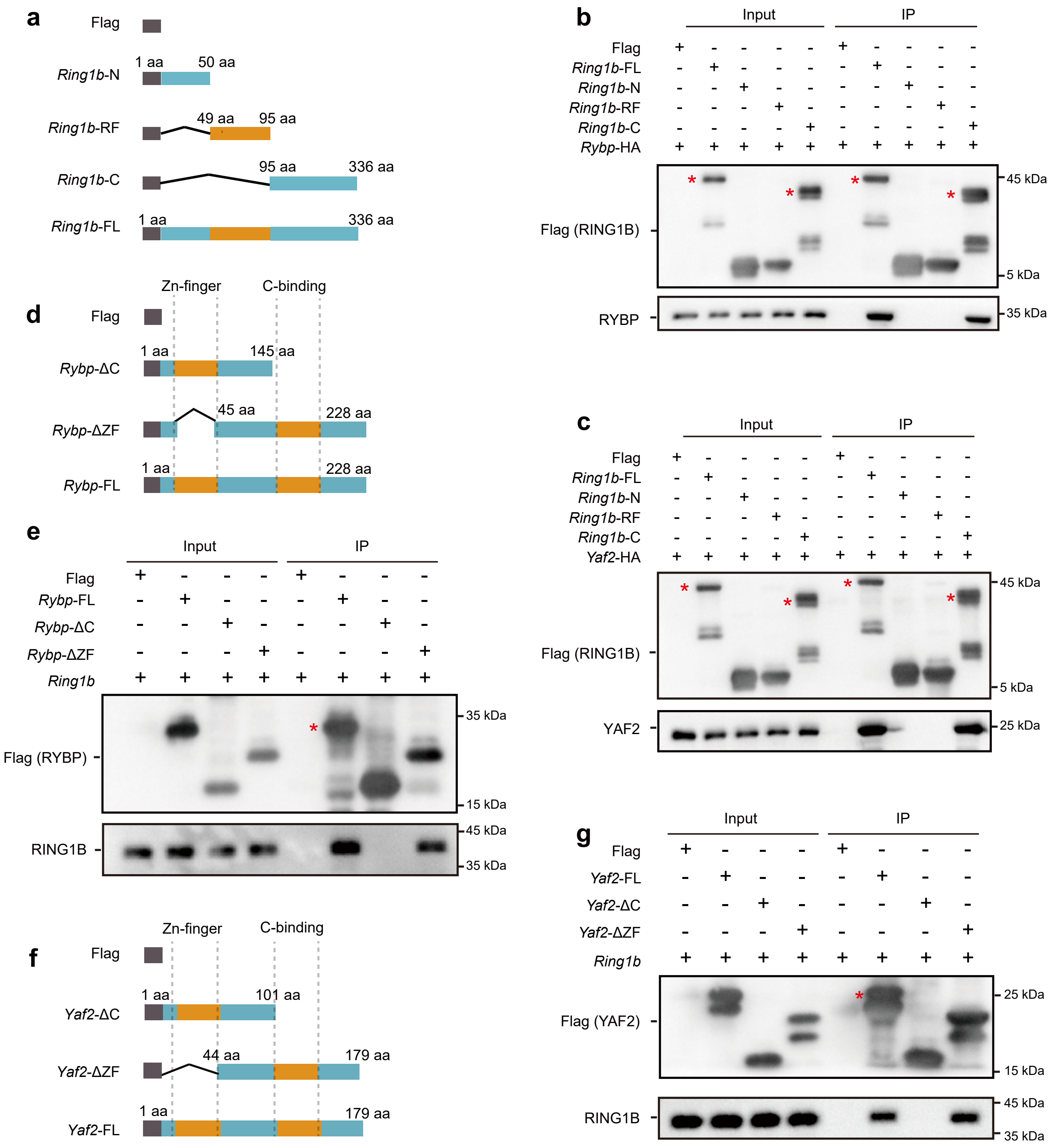

Supplementary Figure 1. RYBP and YAF2 interact with RING1B at the C-terminus, respectively. a Schematic representation of Flag-*Ring1b* and its deletions used in Flag co-IP assays. N: N-terminus; RF: ring finger region; C: C-terminus; FL: full length. **b** Flag immunoprecipitations to detect the interaction between RYBP and Flag-RING1B or Flag-tagged RING1B deletions. Asterisks indicate target proteins. **c** Flag immunoprecipitations to detect the interaction between YAF2 and Flag-RING1B or Flag-tagged RING1B deletions. Asterisks indicate target proteins. **d** Schematic representation of Flag-*Rybp* and its deletions used in Flag co-IP assays. ZF: zinc finger. **e** Detection of the interaction between RING1B and full-length RYBP or its deletions. Asterisks indicate target proteins. **f** Schematic representation of Flag-*Yaf2* and its deletions used in Flag co-IP assays. **g** Detection of the interaction between RING1B and full-length YAF2 or its deletions. Asterisks indicate target proteins. For **b**, **c**, **e** and **g**, representative immunoblots (n = 3 independent experiments) are shown. Source data are provided as a Source Data file.

Supplementary Figure 2

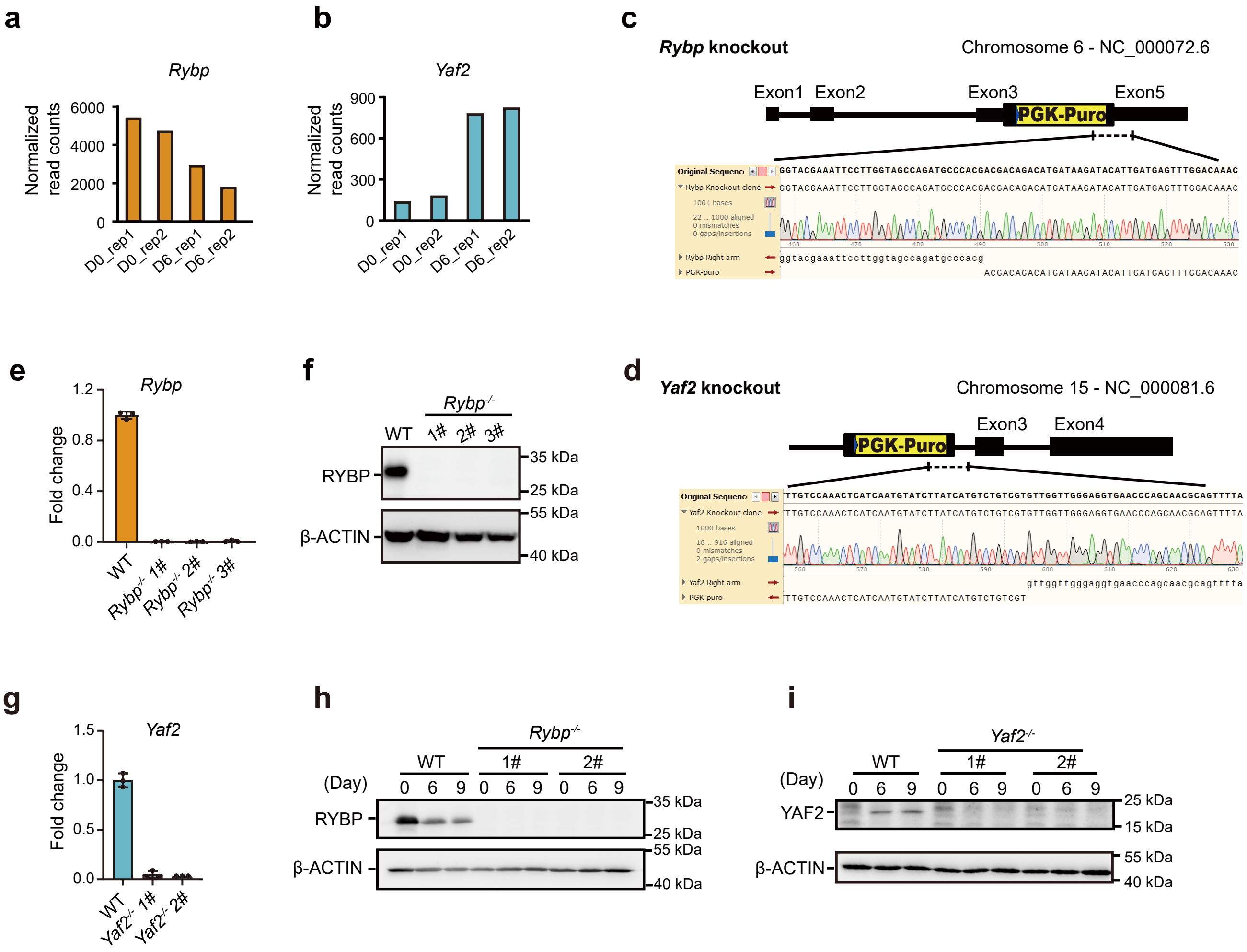

Supplementary Figure 2. Expression of *Rybp* and *Yaf2* during mESC neural differentiation and generation of *Rybp*$^{-/-}$ or *Yaf2*$^{-/-}$ mESCs. a, b Bar plots showing normalized read counts for *Rybp* (**a**) and *Yaf2* (**b**) expression during mESC neural differentiation from our previous RNA-seq data[4]. **c, d** Upper: Schematic overview depicting the targeting strategy for *Rybp* and *Yaf2* loci using CRISPR/Cas9. PGK: human phosphoglycerol kinase promoter; puro: puromycin resistance gene. Bottom: The insert for either *Rybp* knockout or *Yaf2* knockout was confirmed by Sanger sequencing. **e** RT-qPCR analysis of *Rybp* expression in both wild-type and *Rybp*$^{-/-}$ mESCs. The results are shown relative to wild-type (n=3 independent experiments). **f** Western blot analysis of RYBP expression in both wild-type and *Rybp*$^{-/-}$ mESCs. **g** RT-qPCR analysis of *Yaf2* expression in both wild-type and *Yaf2*$^{-/-}$ mESCs. The results are shown relative to wild-type (n=3 independent experiments). **h** Western blot analysis of RYBP expression in both wild-type and *Rybp*$^{-/-}$ mESCs and neural-differentiated cells. **i** Western blot analysis of YAF2 expression in both wild-type and *Yaf2*$^{-/-}$ mESCs and neural-differentiated cells. For **e** and **g**, data are represented as the mean values ± s.d.s. Source data are provided as a Source Data file.

Supplementary Figure 3

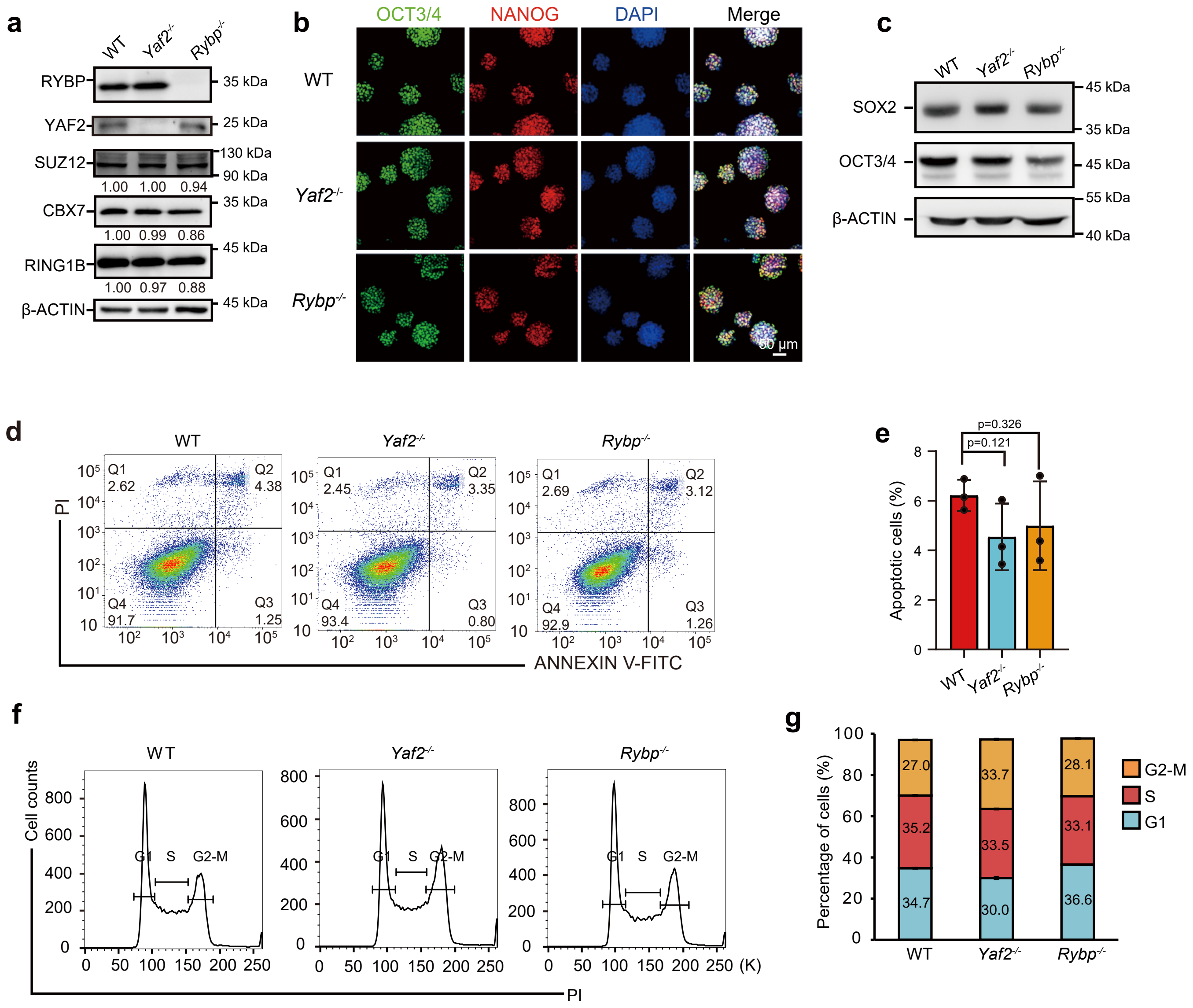

Supplementary Figure 3. Effects of knocking out either *Rybp* or *Yaf2* on apoptosis and cell cycle. a Western blot analysis of RYBP, YAF2, SUZ12, CBX7 and RING1B expression in wild-type, *Rybp*$^{-/-}$ and *Yaf2*$^{-/-}$ mESCs. **b** Immunofluorescence of OCT3/4 (green) and NANOG (red) in wild-type, *Rybp*$^{-/-}$ and *Yaf2*$^{-/-}$ mESCs. DNA was stained with DAPI (blue). Scale bar, 50 µm. **c** Western blot analysis of OCT3/4 and SOX2 expression in wild-type, *Rybp*$^{-/-}$ and *Yaf2*$^{-/-}$ mESCs. **d** FACS analysis of the percentage of apoptotic cells in wild-type, *Rybp*$^{-/-}$ and *Yaf2*$^{-/-}$ mESCs. Annexin V and PI were used to discriminate necrotic cells (Q1), late apoptotic cells (Q2), early apoptotic cells (Q3) and intact cells (Q4). **e** Statistical analysis of the total apoptotic cells, including early apoptotic cells and late apoptotic cells, in panel (**d**) (n=3 independent experiments). **f** FACS analysis of the cell cycle in wild-type, *Rybp*$^{-/-}$ and *Yaf2*$^{-/-}$ mESCs. **g** Statistical analysis of the cell cycle distribution in panel (**f**) (n=3 independent experiments). For **a**-**c**, representative immunoblots (n = 3 independent experiments) are shown. For **e**, data are represented as the mean values ± s.d.s with the indicated significance from two-sided *t* test. Source data are provided as a Source Data file.

Supplementary Figure 4

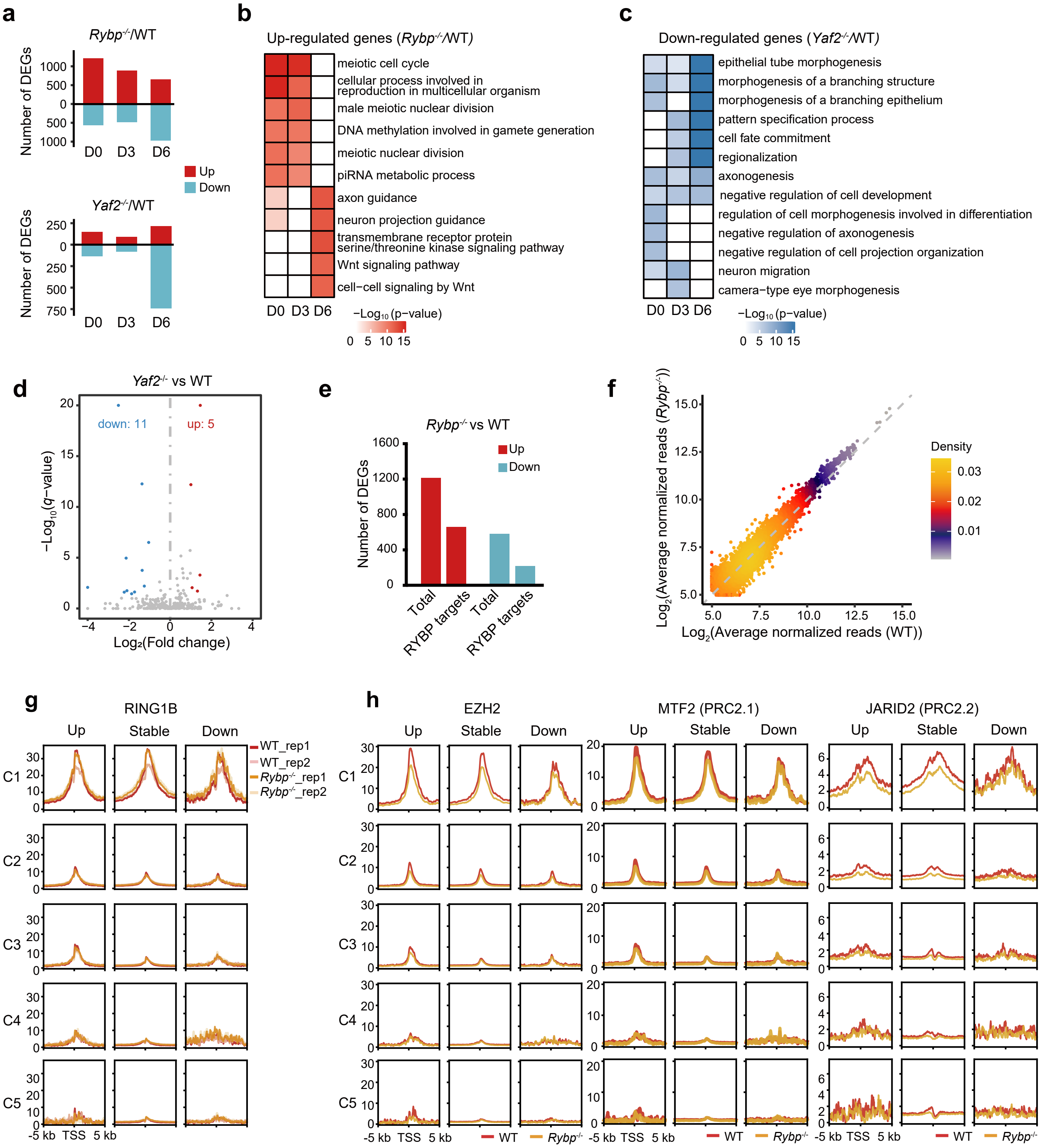

Supplementary Figure 4. Effect of loss of either *Rybp* or *Yaf2* on gene expression and PRC2 binding. a Bar plots showing the number of differentially expressed genes between *Rybp*$^{-/-}$ mESCs and wild-type mESCs (upper) or between *Yaf2*$^{-/-}$ mESCs and wild-type mESCs (bottom) during neural differentiation. **b** GO analysis of the biological functions of upregulated target genes in *Rybp*$^{-/-}$ mESCs relative to wild-type mESCs during neural differentiation. The results are expressed as -Log_{10}(p value). **c** GO analysis of the biological functions of downregulated target genes in *Yaf2*$^{-/-}$ mESCs relative to wild-type mESCs during neural differentiation. The results are expressed as -Log_{10}(p value). **d** Scatter plot showing changes in YAF2 target genes between wild-type and *Yaf2*$^{-/-}$ mESCs. **e** Bar plots showing the number of DEGs among RYBP target genes and total DEGs after *Rybp* knockout in mESCs. **f** Scatter plot showing the comparison of normalized RING1B ChIP signals in wild-type and *Rybp*$^{-/-}$ mESCs. **g** Average enrichment of RING1B around TSS regions for different RYBP target genes between wild-type and *Rybp*$^{-/-}$ mESCs from Fig. 2d. **h** Average enrichment of EZH2, MTF2 and JARID2 around TSS regions for different RYBP target genes between wild-type (red line) and *Rybp*$^{-/-}$ (orange line) mESCs from Fig. 2d. Source data are provided as a Source Data file.

Supplementary Figure 5

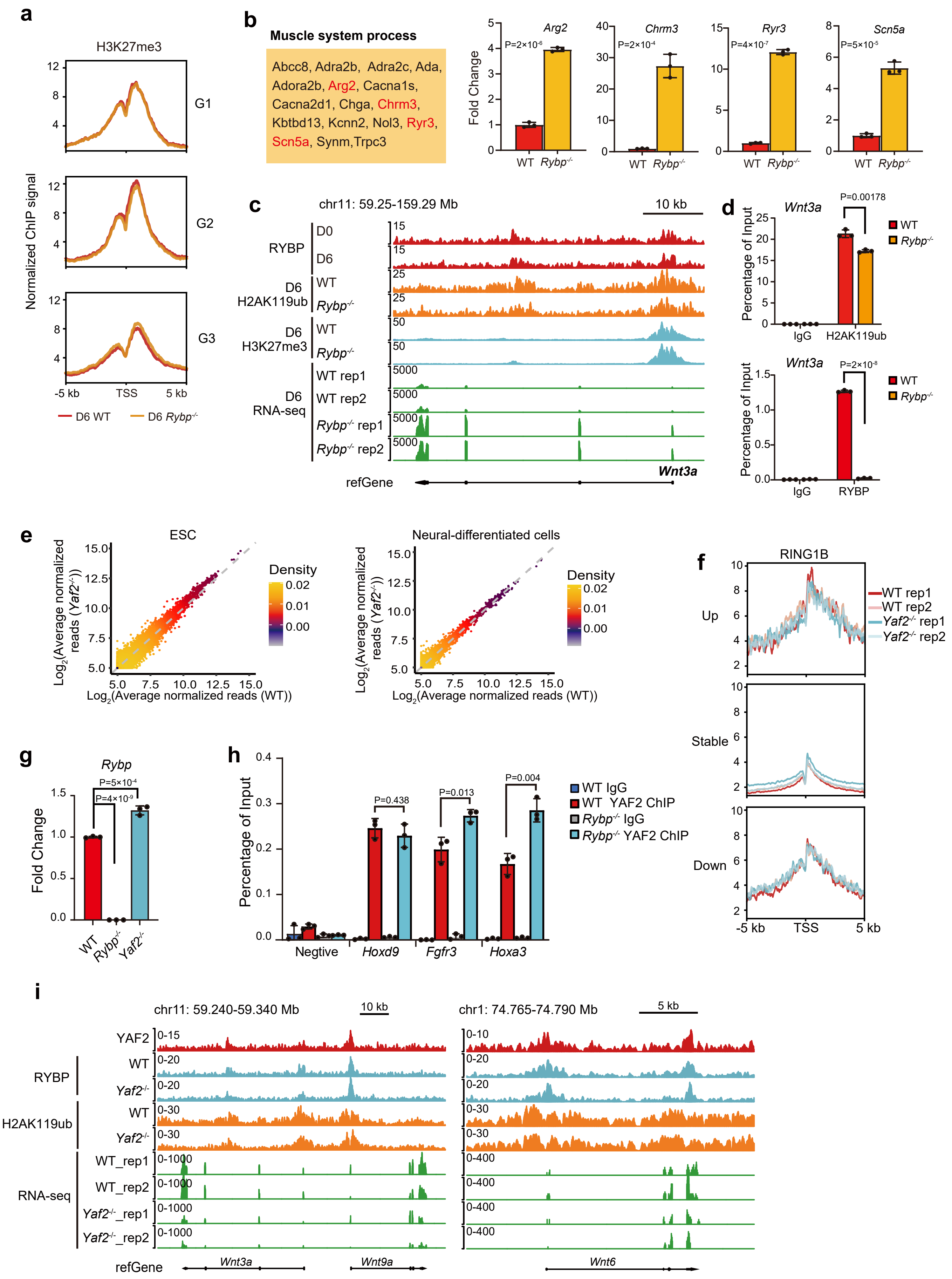

Supplementary Figure 5. Analysis of the derepressed RYBP target genes after loss of either *Rybp* or *Yaf2*. a Average enrichment of H3K27me3 in the promoters of different classes of genes between wild-type and *Rybp*$^{-/-}$ neural-differentiated cells on day 6 from Fig. 3c. **b** RT-qPCR analysis of the expression of muscle-related genes in wild-type and *Rybp*$^{-/-}$ neural differentiated cells on day 3 (n=3 independent experiments). **c** Representative genomic tracks showing the normalized signal for RYBP, H2AK119ub, H3K27me3 and RNA-seq data at the *Wnt3a* gene locus in neural-differentiated cells on day 6. **d** ChIP-qPCR showing relative enrichment of H2AK119ub and RYBP in the promoter region of *Wnt3a* in both wild-type and *Rybp*$^{-/-}$ neural-differentiated cells on day 6. IgG was used as the negative control. (n=3 independent experiments) **e** Scatter plots showing the comparison of normalized RING1B ChIP signals between wild-type and *Yaf2*$^{-/-}$ mESCs (left) and between wild-type and *Yaf2*$^{-/-}$ neural differentiated cells on day 6 (right). **f** Average normalized RING1B ChIP signal in the promoters of upregulated, stable and downregulated YAF2 target genes. **g** RT-qPCR analysis of the expression of *Rybp* in wild-type, *Rybp*$^{-/-}$ and *Yaf2*$^{-/-}$ neural differentiated cells on day 6 (n=3 independent experiments). **h** ChIP-qPCR showing the relative enrichment of YAF2 in the promoter regions of *Hoxd9*, *Fgfr3* and *Hoxa3* in both wild-type and *Rybp*$^{-/-}$ mESCs (n=3 independent experiments). **i** Genomic tracks showing YAF2, RYBP, H2AK119ub and RNA-seq at the *Wnt3a*, *Wnt9a* and *Wnt6* gene loci in wild-type and *Yaf2*$^{-/-}$ neural differentiated cells on day 6. For **b**, **d**, **g** and **h**, data are represented as the mean values ± s.d.s with the indicated significance from two-sided *t* test. Source data are provided as a Source Data file.

Supplementary Table 1. Oligonucleotide information for PCR in this study.

Name	Sequence（5‘-3’）
pSin-*Ring1b* (FL-HA)-EcoRⅠ-F	acaaggatgacgacgataagATGTCTCAGGCTGTGCAGAC
pSin-*Ring1b* (FL-HA)-EcoRⅠ-R	cggccgctttctcgaggccTCAAGCGTAATCTGGAACATCGTATGGGTATTTGTGCTCCTTGGTGG
pSin-*Ring1b* (FL no Flag)-F	gtcgtgaggaattggccaccATGTCTCAGGCTGTGCAGAC
pSin-*Ring1b* (FL no Flag)-R	ggcggccgctttctcgaggccTCATTTGTGCTCCTTGGTGG
pSin-Flag-*Ring1b* (N)-F	cgacgataaggaattcATGTCTCAGGCTGTGC
pSin-Flag-*Ring1b* (N)-R	tatggctagcgaattcTCACATTAATTCACTGTGTAGACTTC
pSin-Flag-*Ring1b* (RF)-F	cgacgataaggaattcTTAATGTGCCCAATTT
pSin-Flag-*Ring1b* (RF)-R	tatggctagcgaattctcaTCAAACCAGTTTTTTCCGACAGG
pSin-Flag-*Ring1b* (C)-F	cgacgataaggaattcGTTTCTAAAAGATCACTAAGGCC
pSin-Flag-*Ring1b* (C)-R	tatggctagcgaattctcaTCATTTGTGCTCCTTGGTGGG
pSin-*Rybp* (FL)-EcoRⅠ-F	atgacgacgataaggaattcATGACCATGGGCGACAAGAA
pSin-*Rybp* (FL)-EcoRⅠ-R	ctcatatggctagcgaattcTCAGAAAGATTCATCATTCA
pSin-*Rybp* (△ZF)- EcoRⅠ-F	atgacgacgataaggaattcGATGTGCGGAAAGGCACCTC
pSin-*Rybp* (△C)- EcoRⅠ-R	ctcatatggctagcgaattcTCACCTTGAGGTGTGGTTTGTTT
pSin-*Rybp* (FL-HA)-EcoRⅠ-F	gtcgtgaggaattggccaccATGACCATGGGCGACAAGAA
pSin-*Rybp* (FL-HA)-EcoRⅠ-R	gcggccgctttctcgaggccTCAAGCGTAATCTGGAACATCGTATGGGTAgaaagattcatcattcactgctg
pSin-*Yaf2* (FL)-EcoRⅠ-F	atgacgacgataaggaattcATGGGCGACAAGAAGAGCCC
pSin-*Yaf2* (FL)-EcoRⅠ-R	ctcatatggctagcgaattcTTAGTGAGACTCTCCGTTGAGGG
pSin-*Yaf2* (△ZF)-EcoRⅠ-F	atgacgacgataaggaattcGTGCGGAAGGGCACCTCCAC
pSin-*Yaf2* (△C)-EcoRⅠ-R	ctcatatggctagcgaattcTTAGGTTTTCTTGTGACAGTTCT
pSin-*Yaf2* (FL-HA)-EcoRⅠ-F	gtcgtgaggaattggccaccATGGGCGACAAGAAGAGCCC
pSin-Yaf2(FL-HA)-EcoRⅠ-R	gcggccgctttctcgaggccTCAAGCGTAATCTGGAACATCGTATGGGTAgtgagactctccgttgagg
pET28a-*Yaf2*-mCherry-F	tgggtcgcggatccgaattcATGGGCGACAAGAAGAGCCC
pET28a-*Yaf2*-mCherry-R	cggatctcattgtttaaacgaTTACTTGTACAGCTCGTCCATGCCG
pGEX4T-GST-*Ring1b*-F	atctggttccgcgtggatccATGTCTCAGGCTGTGCAGAC
pGEX4T-GST-*Ring1b*-R	ggcagatcgtcagtcagtcaTCATTTGTGCTCCTTGGTGGGTG

Supplementary Table 2. sgRNA sequences used in this study.

Name	sequence（5‘-3’）
pX330-*Yaf2*-deletion-F	caccgTATCGCTTGTAGGATCGTAA
pX330-*Yaf2*-deletion-R	aaacTTACGATCCTACAAGCGATAc
pX330-*Rybp*-deletion-F	caccgTGTATGCTGTTAGCTTCACT
pX330-*Rybp*-deletion-R	aaacAGTGAAGCTAACAGCATACAc

supplementary Table 3. Antibody information and catalog number in this study.

REAGENT or RESOURCE	SOURCE	IDENTIFIER	Dilution
Rabbit polyclonal anti-RING1B	Abcam	Cat# ab101273, RRID:AB_10711495	1:1000 for WB
Rabbit polyclonal anti-RING1B	CST	Cat#5694, RRID:AB_10705604	1:100 for ChIP
Rabbit polyclonal anti-RYBP	Millipore	Cat# AB3637, RRID:AB_11213333	1:1000 for WB, 1:100 for ChIP
Rabbit polyclonal anti-YAF2 (for WB)	Bethyl Laboratories	Cat# A303-654A, RRID:AB_11204594	1:1000 for WB
Rabbit polyclonal anti-YAF2 (for ChIP)	Our lab	NA	1:100 for ChIP
Mouse monoclonal anti-FLAG	Sigma-Aldrich	Cat# F1804, RRID:AB_262044	1:1000 for WB
Rabbit polyclonal anti-HA tag	Abcam	Cat# ab9110, RRID:AB_307019	1:1000 for WB
Mouse monoclonal anti-β-ACTIN	Sigma-Aldrich	Cat# A2228, RRID:AB_476697	1:4000 for WB
Rabbit polyclonal anti-TUBB3	Abcam	Cat# ab18207, RRID:AB_444319	1:1000 for WB
Rabbit polyclonal anti-PAX6	Biolegend	Cat# 901301, RRID:AB_2565003	1:1000 for WB, 1:100 for flow cytometry stain
Mouse monoclonal anti-NESTIN	Cell Signaling Technology	Cat# 33475, RRID:AB_2799037	1:1000 for WB, 1:100 for flow cytometry stain
Mouse monoclonal anti-β-Tubulin	RayBiotech	Cat# RM2003, RRID:AB_2773015	1:1000 for WB
Rabbit polyclonal anti-Non-phospho (Active) β-Catenin (Ser33/37/Thr41)	Cell Signaling Technology	Cat# 8814, RRID:AB_11127203	1:1000 for WB
Rabbit monoclonal anti-H2AK119ub	Cell Signaling Technology	Cat# 8240, RRID:AB_10891618	1:100 for ChIP
Rabbit monoclonal anti-H3K27me3	Cell Signaling Technology	Cat# 9733, RRID:AB_2616029	1:100 for ChIP
Rabbit polyclonal anti-H3K27ac	Active Motif	Cat# 39133, RRID:AB_2561016	1:100 for ChIP
Mouse monoclonal anti-OCT3/4	Santa Cruz Biotechnology	Cat# sc-5279, RRID:AB_628051	1:1000 for WB, 1:100 for IF
Rabbit polyclonal anti-NANOG	Novus	Cat# NB100-58842, RRID:AB_2150116	1:100 for IF
Rabbit polyclonal anti-SUZ12	Active Motif	Cat# 39357, RRID:AB_2614929	1:1000 for WB
Rabbit polyclonal anti-CBX7	Abcam	Cat# ab21873, RRID:AB_726005	1:1000 for WB
Rabbit monoclonal anti-SOX2	Cell Signaling Technology	Cat# 23064, RRID:AB_2714146	1:1000 for WB
Rabbit monoclonal anti-EZH2	Cell Signaling Technology	Cat# 5246, RRID:AB_10694683	1:100 for ChIP
Rabbit polyclonal anti-MTF2	Proteintech	Cat# 16208-1-AP, RRID:AB_2147370	1:100 for ChIP
Rabbit monoclonal anti-JARID2	Cell Signaling Technology	Cat# 13594, RRID:AB_2798269	1:100 for ChIP
Goat Anti-Mouse IgG (H+L) HRP secondary antibody	KangChen Bio-tech	Cat# KC-MM-035	1:5000 for WB
Goat Anti-Rabbit IgG (H+L) HRP secondary antibody	KangChen Bio-tech	Cat# KC-RB-035	1:5000 for WB
Goat anti-mouse IgG Alexa 594 secondary antibody	Invitrogen	Cat# A11032, RRID:AB_2534091	1:500 for flow cytometry stain
Goat anti-rabbit IgG Alexa 647 secondary antibody	Invitrogen	Cat# A21245, RRID:AB_141775	1:500 for flow cytometry stain

Supplementary Table 4. Primer sequences used for RT-qPCR in this study.

**Name**	**Foward Primer（5‘-3’）**	**Reverse Primer（5‘-3’）**
Gapdh	AACTTTGGCATTGTGGAAGGGCTCA	TTGGCAGCACCAGTGGATGCAGGGA
Arg2	TCCTCCACGGGCAAATTCC	GCTGGACCATATTCCACTCCTA
Chrm3	CCTCGCCTTTGTTTCCCAAC	TTGAGGAGAAATTCCCAGAGGT
Ryr3	CTGAGCTGGTCCACTTTGTAAA	GAGGTCACCTAATCCCACTTCA
Scn5a	ATGGCAAACTTCCTGTTACCTC	CCACGGGCTTGTTTTTCAGC
Pax6	GCAGATGCAAAAGTCCAGGTG	CAGGTTGCGAAGAACTCTGTTT
Tubb3	ACTTGGAACCTGGAACCATGG	GGCCTGAATAGGTGTCCAAAGG
Map2	GCCAGCCTCAGAACAAACAG	AAGGTCTTGGGAGGGAAGAAC
Nestin	CCCTGAAGTCGAGGAGCTG	CTGCTGCACCTCTAAGCGA
Sox1	GCACACAGCGTTTTCTCGG	ACATCCGACTCCTCTTCCC
Yaf2	CCCTGGGGAAAAAGTATCGCT	CCTGACCCTTTCCGGACATC
Rybp	CAACGTCACCGTCATTATCACAG	CATCATTCACTGCTGACATGTCG
Wnt3a	AATTTGGAGGAATGGTCTCTCGG	CAGCAGGTCTTCACTTCACAG
Wnt6	GCAAGACTGGGGGTTCGAG	CCTGACAACCACACTGTAGGAG
Wnt9a	GGCCCAAGCACACTACAAG	AGAAGAGATGGCGTAGAGGAAA

Supplementary Table 5. Primer sequences used for ChIP-qPCR in this study.

**Name**	**Sequence（5'-3'）**
Wnt3a -F	GGTGACCTTAAGTCTGCGCT
Wnt3a -R	TCTCAGGCCCTAAGTCCTCC
Wnt6 -F	AGAGAGAGTCAAACGGCTGC
Wnt6 -R	TACTGCCAATGCTGAAGGGG
Hoxa3 -F	TGCCCTAACAGTTTGGCGTC
Hoxa3 -R	GCAGCCTGGTCCTAACGTG
Hoxd9 -F	AAAAGGACTGAGTGCTCCGC
Hoxd9 -R	GGAACGAGTTGCACGGGAAT
Fgfr3 -F	TTTAGCGCCTGCTAGGACTG
Fgfr3 -R	GACACCCGACGCAAGAAGTA
Negtive ctrl-F	CTTCTCACGGCCCCAATCAT
Negtive ctrl-R	CATCCCTGTAGCGACCCATC

Supplementary Table 6. Mapping statistic and peak calling information for ChIP-seq data.

IP	Treatment	Cells	Total reads	Mapping ratio	Unique reads	Unique reads (subsample)	Control reads	Number of peaks
RYBP	Wild-type	mESCs	46610491	97.73	26420512	——	27473567	10191
YAF2	Wild-type	mESCs	47948159	97.92	25667850	——	21668926	504
RYBP	Wild-type	neural differentiated cells on day 6	46890923	97.74	29032555	——	20930723	6991
YAF2	Wild-type	neural differentiated cells on day 6	39512031	97.62	25455192	——	20930723	6733
RYBP	*Yaf2* $^{-/-}$	neural differentiated cells on day 6	40420126	97.23	25249341	——	22208382	9742
YAF2	*Rybp* $^{-/-}$	neural differentiated cells on day 6	38118491	97.8	24101282	——	24090025	9020
H2AK119ub	Wild-type	mESCs	49895173	41.44	14179265	14179265	24836635	23152
H2AK119ub	*Rybp* $^{-/-}$	mESCs	29276653	83.19	16660210	16660210	27473567	6510
H2AK119ub	*Yaf2* $^{-/-}$	mESCs	51420275	80.33	29682587	14984156	21668926	17164
H2AK119ub	Wild-type	neural differentiated cells on day 6	34442358	88.99	24625869	14985097	20930723	34029
H2AK119ub	*Rybp* $^{-/-}$	neural differentiated cells on day 6	29067210	86.07	20078291	14984753	24090025	39849
H2AK119ub	*Yaf2* $^{-/-}$	neural differentiated cells on day 6	33948990	85.42	23531078	14983559	22208382	41678
H3K27me3	Wild-type	mESCs	36791122	92.76	23701592	——	24836635	68776
H3K27me3	*Rybp* $^{-/-}$	mESCs	32308684	94.08	20847570	——	27473567	70771
H3K27me3	*Yaf2* $^{-/-}$	mESCs	30648343	93.45	19804866	——	21668926	65625
H3K27me3	Wild-type	neural differentiated cells on day 6	29054469	84.57	17516424	——	20930723	17106
H3K27me3	*Rybp* $^{-/-}$	neural differentiated cells on day 6	40306905	87.4	24413112	——	24090025	18139
H3K27me3	*Yaf2* $^{-/-}$	neural differentiated cells on day 6	29746850	85.35	17781997	——	22208382	16140
EZH2	Wild-type	mESCs	37154383	90.31	17685477	——	24836635	10403
EZH2	*Rybp* $^{-/-}$	mESCs	51726087	91.12	22917974	——	27473567	6261
EZH2	*Yaf2* $^{-/-}$	mESCs	44744908	90.45	21079457	——	21668926	8293
MTF2	Wild-type	mESCs	35083775	90.07	17421943	——	24836635	5258
MTF2	*Rybp* $^{-/-}$	mESCs	55362595	89.79	24695369	——	27473567	6386
MTF2	*Yaf2* $^{-/-}$	mESCs	53795477	89.69	24616676	——	21668926	6264
JARID2	Wild-type	mESCs	30333656	80.08	13623305	——	24836635	2833
JARID2	*Rybp* $^{-/-}$	mESCs	35353350	65.33	11911870	——	27473567	2615
JARID2	*Yaf2* $^{-/-}$	mESCs	36593547	89.36	20491309	——	21668926	4995
H3K27ac	Wild-type	mESCs	40832081	94.3	21452788	——	24836635	33998
H3K27ac	*Rybp* $^{-/-}$	mESCs	42250404	93.78	21132180	——	27473567	31015
RING1B_rep1	Wild-type	mESCs	38203769	86.2	17190604	10000000	24836635	12282
RING1B_rep2	Wild-type	mESCs	31620588	81.3	12939006	10000000	24836635	10104
RING1B_rep1	*Rybp* $^{-/-}$	mESCs	47161455	85.9	20465203	10000000	27473567	12349
RING1B_rep2	*Rybp* $^{-/-}$	mESCs	27849080	80.7	11402658	10000000	27473567	7900
RING1B_rep1	*Yaf2* $^{-/-}$	mESCs	38946166	87	18129307	10000000	21668926	11181
RING1B_rep2	*Yaf2* $^{-/-}$	mESCs	29074955	80.3	11395642	10000000	21668926	6735
RING1B_rep1	Wild-type	neural differentiated cells on day 6	42970425	86.6	19518458	10000000	20930723	6453
RING1B_rep2	Wild-type	neural differentiated cells on day 6	27827564	81.2	11452011	10000000	20930723	3945
RING1B_rep1	*Rybp* $^{-/-}$	neural differentiated cells on day 6	38115125	85.2	17092121	10000000	24090025	7793
RING1B_rep2	*Rybp* $^{-/-}$	neural differentiated cells on day 6	30223096	79.9	11591762	10000000	24090025	4619
RING1B_rep1	*Yaf2* $^{-/-}$	neural differentiated cells on day 6	38730079	86	17578510	10000000	22208382	3760
RING1B_rep2	*Yaf2* $^{-/-}$	neural differentiated cells on day 6	30831114	79.6	11860033	10000000	22208382	3477

Supplementary Table 7. Normalization information for calibrated ChIP-seq data.

IP	Treatment	Cells	Reads mapped to mm10	Reads mapped to dm6	dm6/mm10	Scale factor
H2AK119ub	Wild-type	mESCs	14160715	14682	0.00103681	1
H2AK119ub	*Rybp* $^{-/-}$	mESCs	16645528	18550	0.00111441	0.930365718
H2AK119ub_subsample	*Yaf2* $^{-/-}$	mESCs	14984157	15843	0.00105732	0.980606867
H2AK119ub_subsample	Wild-type	neural differentiated cells on day 6	14985097	14903	0.00099452	1.042523599
H2AK119ub_subsample	*Rybp* $^{-/-}$	neural differentiated cells on day 6	14984753	15247	0.0010175	1.018978982
H2AK119ub_subsample	*Yaf2* $^{-/-}$	neural differentiated cells on day 6	14983559	16441	0.00109727	0.944902048
H3K27me3	Wild-type	mESCs	23701592	24848	0.00104837	1
H3K27me3	*Rybp* $^{-/-}$	mESCs	20847570	20270	0.0009723	1.078240431
H3K27me3	*Yaf2* $^{-/-}$	mESCs	19804866	22152	0.00111851	0.937287631
H3K27me3	Wild-type	neural differentiated cells on day 6	17516424	88591	0.0050576	0.207285902
H3K27me3	*Rybp* $^{-/-}$	neural differentiated cells on day 6	24413112	129641	0.0053103	0.197421611
H3K27me3	*Yaf2* $^{-/-}$	neural differentiated cells on day 6	17781997	85738	0.00482162	0.21743082

IP	Treatment	Cells	Reads mapped to mm10	Reads mapped to hg38	hg38/mm10	Scale factor
EZH2	Wild-type	mESCs	35370954	2355352	0.06659	1
EZH2	*Rybp* $^{-/-}$	mESCs	45835948	4101678	0.08948605	0.744138375
EZH2	*Yaf2* $^{-/-}$	mESCs	42158914	3321154	0.07877703	0.845297234
MTF2	Wild-type	mESCs	34843886	2519624	0.0723118	1
MTF2	*Rybp* $^{-/-}$	mESCs	49390738	4611920	0.09337621	0.713136716
MTF2	*Yaf2* $^{-/-}$	mESCs	49233352	5723928	0.11626119	0.572762123
JARID2	Wild-type	mESCs	27246610	1295582	0.04755021	1
JARID2	*Rybp* $^{-/-}$	mESCs	23823740	1580470	0.06634013	0.716763884
JARID2	*Yaf2* $^{-/-}$	mESCs	40982618	2384156	0.05817481	0.817367677

Supplementary Table 8. Mapping statistic and peak calling information for ATAC-seq data.

Sample	Treatment	Cells	Total reads	Mapping ratio	Unique reads	Number of peaks
Replicate 1	Wild-type	mESCs	30790196	97.59	17090337	49139
Replicate 2	Wild-type	mESCs	30527311	97.52	17533725	46610
Replicate 1	*Rybp* $^{-/-}$	mESCs	36677783	97.46	19305413	61943
Replicate 2	*Rybp* $^{-/-}$	mESCs	33560589	97.29	19305055	55007
