# Supplementary material for: Functional dissection of PRC1 subunits RYBP and YAF2 during neural differentiation of embryonic stem cells
Source: Nat Commun. 2023 Nov 7;14:7164. doi: 10.1038/s41467-023-42507-9 (PMC10630410; doi:10.1038/s41467-023-42507-9)
Supplement: Supplementary file 2 — Reporting Summary [file 41467_2023_42507_MOESM2_ESM.pdf]

nature portfolio

Corresponding author(s): Hongjie Yao

Last updated by author(s): Oct 8, 2023

Reporting Summary

Nature Portfolio wishes to improve the reproducibility of the work that we publish. This form provides structure for consistency and transparency in reporting. For further information on Nature Portfolio policies, see our Editorial Policies and the Editorial Policy Checklist.

Statistics

For all statistical analyses, confirm that the following items are present in the figure legend, table legend, main text, or Methods section.

n/a	Confirmed	
☐	☒	The exact sample size (*n*) for each experimental group/condition, given as a discrete number and unit of measurement
☐	☒	A statement on whether measurements were taken from distinct samples or whether the same sample was measured repeatedly
☐	☒	The statistical test(s) used AND whether they are one- or two-sided<br>*Only common tests should be described solely by name; describe more complex techniques in the Methods section.*
☒	☐	A description of all covariates tested
☒	☐	A description of any assumptions or corrections, such as tests of normality and adjustment for multiple comparisons
☐	☒	A full description of the statistical parameters including central tendency (e.g. means) or other basic estimates (e.g. regression coefficient) AND variation (e.g. standard deviation) or associated estimates of uncertainty (e.g. confidence intervals)
☐	☒	For null hypothesis testing, the test statistic (e.g. *F*, *t*, *r*) with confidence intervals, effect sizes, degrees of freedom and *P* value noted<br>*Give P values as exact values whenever suitable.*
☒	☐	For Bayesian analysis, information on the choice of priors and Markov chain Monte Carlo settings
☒	☐	For hierarchical and complex designs, identification of the appropriate level for tests and full reporting of outcomes
☒	☐	Estimates of effect sizes (e.g. Cohen's *d*, Pearson's *r*), indicating how they were calculated

Our web collection on statistics for biologists contains articles on many of the points above.

Software and code

Policy information about availability of computer code

Data collection	RNA-seq, ChIP-seq and ATAC-seq libraries were sequenced on Illumina NovaSeq 6000. BD LSRFortessa was used for Flow cytometry.
Data analysis	Image J was used for quantification of signals from Western blot. FlowJo (v10) was used to analyze FACS data. Data was plotted using GraphPad Prism (v8.0.2). Adobe Illustrator 2020 was used to prepare figures.<br>For RNA-seq, the raw paired end reads were trimmed using Trim_Galore (v0.6.5), then mapped and quantified to mouse mm10 genome with STAR-RSEM pipeline using RSEM (v1.2.22). Transcript-level counts were collapsed to gene-level counts using tximport (v1.20.0). DESeq2 (v1.32.0) was used for finding differential genes. GO analysis was conducted with clusterProfiler (v4.0.0).<br>Bowtie2 (v2.2.5) was used for alignment ChIP-seq data. Peak annotation was performed with ChIPseeker (v1.28.3). bamCoverage (v3.5.1) from deepTools was used to generate the normalized bigwig files. Accessible regions were identified using MACS2 (v2.2.7.1) without control using the default parameters, and differential analysis was performed using DiffBind (v3.2.4). Adaptors and low-quality reads were trimmed with Trim_Galore (v0.6.5). For RYBP, YAF2, RING1B and H3K27ac ChIP-seq, the trimmed reads were aligned to the mouse mm10 genome using bowtie2 (v2.2.5) with the parameter "--very-sensitive --end-to-end --no-unal". For H2AK119ub and H3K27me3 ChIP-seq, the trimmed reads were mapped to mm10 (mouse) and dm6 (drosophila melanogaster) reference genomes using bowtie2 (v2.2.5) with the parameters "--very-sensitive --end-to-end --no-unal --no-mixed --no-discordant". For EZH2, JARID2 and MTF2 ChIP-seq, the trimmed reads were mapped to mm10 (mouse) and hg38 (human) reference genomes using bowtie2 (v2.2.5) with the same parameters as above. The mapped reads with a quality lower than 30 were filtered out. Duplicated reads were removed with sambamba (v0.6.7). The reads overlapping with mouse mm10 blacklist regions (http://mitra.stanford.edu/kundaje/akundaje/release/blacklists) were excluded.For RING1B ChIP-seq, uniquely mapped reads for each sample were subsampled to 10 million, and replicates were subjected to DiffBind (v3.2.4) for differential binding analysis. For calibrated ChIP-seq (cChIP-seq), the reads mapped to mm10, dm6 or hg38 genome were extracted and calculated for calibration as previously described63. bamCoverage (v3.5.1) from deepTools was used to generate the normalized bigwig files with the parameter "--normalizeUsing RPGC" for normal ChIP-seq and "--normalizeUsing RPGC --scaleFactor" for cChIP-seq. The reads mapped to mm10 genome were used to

identify peaks by MACS2 (v2.2.7.1) in broad mode with the default parameters. Peak annotation was performed with ChIPseeker (v1.28.3). Target genes of both RYBP and YAF2 were identified when they bound within 1 kb of the transcription start sites (TSSs).

For ATAC-seq, paired-end reads were treated with trim_galore (v0.6.5) to remove adapters and low-quality reads and then aligned to the mm10 by using bowtie2 (v2.2.5) with the parameters "--very-sensitive --end-to-end --no-unal -X 2000". The alignment files were further processed with sambamba (v0.6.7) to remove low-quality mapped reads and duplicates. The reads overlapping with mouse mm10 blacklist regions (http://mitra.stanford.edu/kundaje/akundaje/release/blacklists) were filtered out. Accessible regions were identified using MACS2 (v2.2.7.1) without control using the default parameters, and differential analysis was performed using DiffBind (v3.2.4). Normalized bigWig files were generated using bamCoverage (v3.5.1).

For manuscripts utilizing custom algorithms or software that are central to the research but not yet described in published literature, software must be made available to editors and reviewers. We strongly encourage code deposition in a community repository (e.g. GitHub). See the Nature Portfolio guidelines for submitting code & software for further information.

Data

Policy information about availability of data

All manuscripts must include a data availability statement. This statement should provide the following information, where applicable:

- Accession codes, unique identifiers, or web links for publicly available datasets
- A description of any restrictions on data availability
- For clinical datasets or third party data, please ensure that the statement adheres to our policy

The ATAC-seq, ChIP-seq and RNA-seq data reported in this paper have been deposited in the Genome Sequence Archive database in the National Genomics Data Center (GSA: CRA006987) [https://ngdc.cncb.ac.cn/gsa/browse/CRA006987]and in the Gene Expression Omnibus (GEO) database (GSE213416) [https://www.ncbi.nlm.nih.gov/geo/query/acc.cgi?&acc=GSE213416]. Source data are provided with this paper. Any additional information required to reanalyze the data reported in this paper is available from the lead contact upon request.

Human research participants

Policy information about studies involving human research participants and Sex and Gender in Research.

Reporting on sex and gender	*Use the terms sex (biological attribute) and gender (shaped by social and cultural circumstances) carefully in order to avoid confusing both terms. Indicate if findings apply to only one sex or gender; describe whether sex and gender were considered in study design whether sex and/or gender was determined based on self-reporting or assigned and methods used. Provide in the source data disaggregated sex and gender data where this information has been collected, and consent has been obtained for sharing of individual-level data; provide overall numbers in this Reporting Summary. Please state if this information has not been collected. Report sex- and gender-based analyses where performed, justify reasons for lack of sex- and gender-based analysis.*
Population characteristics	*Describe the covariate-relevant population characteristics of the human research participants (e.g. age, genotypic information, past and current diagnosis and treatment categories). If you filled out the behavioural & social sciences study design questions and have nothing to add here, write "See above."*
Recruitment	*Describe how participants were recruited. Outline any potential self-selection bias or other biases that may be present and how these are likely to impact results.*
Ethics oversight	*Identify the organization(s) that approved the study protocol.*

Note that full information on the approval of the study protocol must also be provided in the manuscript.

Field-specific reporting

Please select the one below that is the best fit for your research. If you are not sure, read the appropriate sections before making your selection.

☒ Life sciences ☐ Behavioural & social sciences ☐ Ecological, evolutionary & environmental sciences

For a reference copy of the document with all sections, see nature.com/documents/nr-reporting-summary-flat.pdf

Life sciences study design

All studies must disclose on these points even when the disclosure is negative.

Sample size	At least three independent replicates were used in each experiment, and the replicate number was presented in the figure legends.
Data exclusions	None
Replication	The results were repeated at least three times as independent replicates.
Randomization	N/A

Blinding	There were no human or animal research participants involved in this study. Blinding is not relevant.

Reporting for specific materials, systems and methods

We require information from authors about some types of materials, experimental systems and methods used in many studies. Here, indicate whether each material, system or method listed is relevant to your study. If you are not sure if a list item applies to your research, read the appropriate section before selecting a response.

Materials & experimental systems

n/a	Involved in the study
☐	☒ Antibodies
☐	☒ Eukaryotic cell lines
☒	☐ Palaeontology and archaeology
☒	☐ Animals and other organisms
☒	☐ Clinical data
☒	☐ Dual use research of concern

Methods

n/a	Involved in the study
☐	☒ ChIP-seq
☐	☒ Flow cytometry
☒	☐ MRI-based neuroimaging

Antibodies

Antibodies used	Primary antibodies uesd were: Rabbit polyclonal anti-RING1B (Abcam, Cat#ab101273, dilution 1:1000 for WB), Rabbit polyclonal anti-RING1B (CST, Cat#5694, Lot#4, dilution 1:100 for ChIP), Rabbit polyclonal anti-RYBP (Millipore, Cat#AB3637, dilution 1:1000 for WB, 1:100 for ChIP), Rabbit polyclonal anti-YAF2 (Bethyl Laboratories, Cat#A303-654A, dilution 1:1000 for WB) , Rabbit polyclonal anti-YAF2 (Our lab,dilution 1:100 for ChIP) , Mouse monoclonal anti-FLAG (Sigma-Aldrich, Cat#F1804, Lot#SLBW5142, dilution 1:1000 for WB), Rabbit polyclonal anti-HA tag (Abcam, Cat#ab9110, dilution 1:1000 for WB), Mouse monoclonal anti-β-ACTIN (Sigma-Aldrich, Cat#A2228, Lot#0000165975, dilution 1:4000 for WB), Rabbit polyclonal anti-TUBB3 (Abcam, Cat#ab18207, dilution 1:1000 for WB), Rabbit polyclonal anti-PAX6 (Biolegend, Cat#901301,dilution 1:1000 for WB, 1:100 for flow cytometry stain), Mouse monoclonal anti-NESTIN (Cell Signaling Technology, Cat#33475, dilution 1:100 for flow cytometry stain), Mouse monoclonal anti-β-Tubulin (RayBiotech, Cat#RM2003, Lot#Z0219, dilution 1:1000 for WB), Rabbit polyclonal anti-Non-phospho (Active) β-Catenin (Ser33/37/Thr41) (Cell Signaling Technology, Cat#8814, Lot#6, dilution 1:1000 for WB), Rabbit monoclonal anti-H2AK119ub (Cell Signaling Technology, Cat#8240, Lot#9, dilution 1:100 for ChIP), Rabbit monoclonal anti-H3K27me3 (Cell Signaling Technology, Cat#9733, Lot#19, dilution 1:100 for ChIP), Rabbit polyclonal anti-H3K27ac (Active Motif, Cat#39133, Lot#31521015, dilution 1:100 for ChIP), Mouse monoclonal anti-OCT3/4 (Santa Cruz Biotechnology, Cat#sc-5279, Lot#H0409, dilution 1:1000 for WB, 1:100 for IF), Rabbit polyclonal anti-NANOG (Novus, Cat#NB100-58842, Lot#A3, dilution 1:100 for IF), Rabbit polyclonal anti-SUZ12 (Active Motif, Cat#39357, dilution 1:1000 for WB), Rabbit polyclonal anti-CBX7 (Abcam, Cat#ab21873, dilution 1:1000 for WB), Rabbit monoclonal anti-SOX2 (Cell Signaling Technology, Cat#23064, Lot#1, dilution 1:1000 for WB), Rabbit monoclonal anti-EZH2 (Cell Signaling Technology, Cat#5246, Lot#10, dilution 1:100 for ChIP), Rabbit polyclonal anti-MTF2 (Proteintech, Cat#16208-1-AP, Lot#00077384, dilution 1:100 for ChIP), Rabbit monoclonal anti-JARID2 (Cell Signaling Technology, Cat#13594, dilution 1:100 for ChIP), Normal Rabbit IgG (Cell Signaling Technology, Cat#2729, dilution 1:100 for ChIP).<br>Secondary antibody used were: Goat Anti-Mouse IgG (H+L) HRP (Cat# KC-MM-035, dilution 1:5000 for WB), Goat anti-Rabbit IgG (H&L) HRP (Cat# KC-RB-035, dilution 1:5000 for WB), goat anti-mouse IgG Alexa 594 secondary antibody (Invitrogen, Cat# A11032, Lot#2527968, dilution 1:500 for IF), goat anti-rabbit IgG Alexa 647 secondary antibody (Invitrogen, Cat#21245, Lot#2390714, dilution 1:500 for IF).
Validation	Rabbit polyclonal anti-YAF2 (Our lab) for ChIP experiments was validated with Wild-type and Yaf2 knockout cell lines. The commercial antibodies were validated by following the manufacturer's methods.

Eukaryotic cell lines

Policy information about cell lines and Sex and Gender in Research

Cell line source(s)	46C mESCs were described in (Ying et al., 2003), RYBP-KO 46C mESCs were described in (Liu et al., 2019) and referred in the manuscript. The HEK293T cell lines was from ATCC and maintained in our lab.
Authentication	Routine quality control was measured with microscopy morphology, and qRT-PCR was performed to test the expression of marker genes.
Mycoplasma contamination	All cell lines were confirmed negative for Mycoplasma contamination.
Commonly misidentified lines (See ICLAC register)	No commonly misidentified line was used.

ChIP-seq

Data deposition

☒ Confirm that both raw and final processed data have been deposited in a public database such as GEO.

☐ Confirm that you have deposited or provided access to graph files (e.g. BED files) for the called peaks.

Data access links<br>*May remain private before publication.*	ChIP-seq reported in this paper have been deposited in the Genome Sequence Archive in National Genomics Data Center, China National Center for Bioinformation/Beijing Institute of Genomics, Chinese Academy of Sciences (GSA: CRA006987) that are publicly accessible at https://ngdc.cncb.ac.cn/gsa and in the Gene Expression Omnibus (GEO) database (GSE213416).
Files in database submission	NPC_input_YAF2_R1.fq.gz<br>NPC_input_YAF2_R2.fq.gz<br>NPC_input_RYBP_R1.fq.gz<br>NPC_input_RYBP_R2.fq.gz<br>NPC_input_46C_R1.fq.gz<br>NPC_input_46C_R2.fq.gz<br>mES_input_YAF2_R1.fq.gz<br>mES_input_YAF2_R2.fq.gz<br>mES_input_RYBP_R1.fq.gz<br>mES_input_RYBP_R2.fq.gz<br>mES_input_46C_R1.fq.gz<br>mES_input_46C_R2.fq.gz<br>mES_RYBP_H3K27ac_R1.fq.gz<br>mES_RYBP_H3K27ac_R2.fq.gz<br>mES_46C_H3K27ac_R1.fq.gz<br>mES_46C_H3K27ac_R2.fq.gz<br>NPC_RybpKO_YAF2_R1.fq.gz<br>NPC_RybpKO_YAF2_R2.fq.gz<br>NPC_WT_YAF2_R1.fq.gz<br>NPC_WT_YAF2_R2.fq.gz<br>NPC_Yaf2KO_RYBP_R1.fq.gz<br>NPC_Yaf2KO_RYBP_R2.fq.gz<br>NPC_WT_RYBP_R1.fq.gz<br>NPC_WT_RYBP_R2.fq.gz<br>mES_YAF2_R1.fq.gz<br>mES_YAF2_R2.fq.gz<br>mES_RYBP_R1.fq.gz<br>mES_RYBP_R2.fq.gz<br>JARID2_ESC_RKO_R1.fq.gz<br>JARID2_ESC_RKO_R2.fq.gz<br>JARID2_ESC_WT_R1.fq.gz<br>JARID2_ESC_WT_R2.fq.gz<br>MTF2_ESC_RKO_R1.fq.gz<br>MTF2_ESC_RKO_R2.fq.gz<br>MTF2_ESC_WT_R1.fq.gz<br>MTF2_ESC_WT_R2.fq.gz<br>EZH2_ESC_RKO_R1.fq.gz<br>EZH2_ESC_RKO_R2.fq.gz<br>EZH2_ESC_WT_R1.fq.gz<br>EZH2_ESC_WT_R2.fq.gz<br>H3K27me3_NPC_YKO_R1.fq.gz<br>H3K27me3_NPC_YKO_R2.fq.gz<br>H3K27me3_NPC_RKO_R1.fq.gz<br>H3K27me3_NPC_RKO_R2.fq.gz<br>H3K27me3_NPC_WT_R1.fq.gz<br>H3K27me3_NPC_WT_R2.fq.gz<br>H3K27me3_46C_YKO_R1.fq.gz<br>H3K27me3_46C_YKO_R2.fq.gz<br>H3K27me3_46C_RKO_R1.fq.gz<br>H3K27me3_46C_RKO_R2.fq.gz<br>H3K27me3_46C_WT_R1.fq.gz<br>H3K27me3_46C_WT_R2.fq.gz<br>D6_YKO_H2AK119ub_R1.fq.gz<br>D6_YKO_H2AK119ub_R2.fq.gz<br>D6_RKO_H2AK119ub_R1.fq.gz<br>D6_RKO_H2AK119ub_R2.fq.gz

	D6_WT_H2AK119ub_R1.fq.gz<br>D6_WT_H2AK119ub_R2.fq.gz<br>mES_YKO_H2AK119ub_R1.fq.gz<br>mES_YKO_H2AK119ub_R2.fq.gz<br>mES_RKO_H2AK119ub_R1.fq.gz<br>mES_RKO_H2AK119ub_R2.fq.gz<br>mES_WT_H2AK119ub_R1.fq.gz<br>mES_WT_H2AK119ub_R2.fq.gz<br>RING1B_NPC_YKO_R1.fq.gz<br>RING1B_NPC_YKO_R2.fq.gz<br>RING1B_NPC_RKO_R1.fq.gz<br>RING1B_NPC_RKO_R2.fq.gz<br>RING1B_NPC_WT_R1.fq.gz<br>RING1B_NPC_WT_R2.fq.gz<br>RING1B_ESC_YKO_R1.fq.gz<br>RING1B_ESC_YKO_R2.fq.gz<br>RING1B_ESC_RKO_R1.fq.gz<br>RING1B_ESC_RKO_R2.fq.gz<br>RING1B_ESC_WT_R1.fq.gz<br>RING1B_ESC_WT_R2.fq.gz
Genome browser session<br>(e.g. UCSC)	BW files were visualized in IGV and the reference genome assembly was Mouse GRCm38/mm10.

Methodology

Replicates	The ChIP experiments for RING1B were repeated twice as experimental replicates.<br>Drosophia melanogaster chromatin was added as spike-in to calibrate the histone modification ChIP-seq data.
Sequencing depth	All above sequencing data were at least 30 M reads; uniquely reads were at least 10 M. Detailed mapping statistic and peak calling information for ChIP-seq data were described in Supplemental Table 6.
Antibodies	Rabbit polyclonal anti-RYBP (Millipore, Cat#AB3637), Rabbit polyclonal anti-YAF2 (Our lab) , Rabbit monoclonal anti-H2AK119ub (Cell Signaling Technology, Cat#8240), Rabbit monoclonal anti-H3K27me3 (Cell Signaling Technology, Cat#9733), Rabbit polyclonal anti-H3K27ac (Active Motif, Cat#39133), Rabbit monoclonal anti-EZH2 (Cell Signaling Technology, Cat#5246), Rabbit polyclonal anti-MTF2 (Proteintech, Cat#16208-1-AP), Rabbit monoclonal anti-JARID2 (Cell Signaling Technology, Cat#13594), Rabbit monoclonal anti-RING1B (Cell Signaling Technology, Cat#5694).
Peak calling parameters	The reads mapped to mm10 genome were used to identify peaks by MACS2 (v2.2.7.1) in broad mode with the default parameters.
Data quality	The mapped reads with the quality lower than 30 were filtered out. Detailed mapping ratio and data quality information were described in Supplementary Table 6.
Software	For RYBP, YAF2 and H3K27ac ChIP-seq, the trimmed reads were aligned to mouse mm10 genome using bowtie2 with parameter "--very-sensitive --end-to-end --no-unal". For H2AK119ub and H3K27me3 ChIP-seq, the trimmed reads were mapped to mm10 (mouse) and dm6 (drosophila melanogaster) reference genome using bowtie2 with parameters "--very-sensitive --end-to-end --no-unal --no-mixed --no-discordant". For EZH2, JARID2 and MTF2 ChIP-seq, the trimmed reads were mapped to mm10 (mouse) and hg38 (human) reference genome using bowtie2 with the same parameters as above. The mapped reads with a quality lower than 30 were filtered out. Duplicated reads were removed with sambamba (v0.6.7). The reads overlapping with mouse mm10 blacklist regions (http://mitra.stanford.edu/kundaje/akundaje/release/blacklists) were excluded.<br>For RING1B ChIP-seq, uniquely mapped reads for each sample were subsampled to 10 million, and replicates were subjected to DiffBind (v3.2.4) for differential binding analysis. For calibrated ChIP-seq (cChIP-seq), the reads mapped to mm10, dm6 or hg38 genome were extracted and calculated for calibration as previously described. bamCoverage (v3.5.1) from deepTools was used to generate the normalized bigwig files with the parameter "--normalizeUsing RPGC" for normal ChIP-seq and "--normalizeUsing RPGC --scaleFactor" for cChIP-seq. The reads mapped to mm10 genome were used to identify peaks by MACS2 (v2.2.7.1) in broad mode with the default parameters. Peak annotation was performed with ChIPseeker (v1.28.3).

Flow Cytometry

Plots

Confirm that:

- [x] The axis labels state the marker and fluorochrome used (e.g. CD4-FITC).
- [x] The axis scales are clearly visible. Include numbers along axes only for bottom left plot of group (a 'group' is an analysis of identical markers).
- [x] All plots are contour plots with outliers or pseudocolor plots.
- [x] A numerical value for number of cells or percentage (with statistics) is provided.

Methodology

Sample preparation

mESCs were dissociated by using 0.25% Trypsin-EDTA (except apoptosis assay without EDTA) and quenched with serum-medium. Day 6 differentiated cells were collected with Accutase (STEM CELL, 7920). Then the cells were prepared for apoptosis by using the Apoptosis Detection Kit (Vazyme Biotech A211-02), and analyzed for cell cycle detection with Cell Cycle Detection Kit (KeyGen, KGA512). To evaluate the neural differentiation efficiency, the cells were fixed with 4% paraformaldehyde at room temperature (RT) for 15 min and permeabilized with 0.3% Triton-100 at RT for 15 min. Then the cells were stained with a mixture of anti-rabbit PAX6 antibody (Biolegend, 901301, dilution 1:100) and anti-mouse NESTIN antibody (CST, 33475, dilution 1:100) a 1:100 dilution at RT for 1hr. After washing with the ice-cold PBS twice, the cells were stained with a mixture of goat anti-mouse IgG Alexa 594 secondary antibody (Invitrogen, cat# A11032, dilution 1:500) and goat anti-rabbit IgG Alexa 647 secondary antibody (Invitrogen, cat#21245,dilution 1:500) at RT for 1hr. Then the cells were washed with ice-cold PBS twice and resuspended in single cell suspension in FACS buffer (DPBS containing 5% FBS), and were passed through a cell strainer (70 μm).

Instrument

LSR Fortessa SORP

Software

FlowJo v.10 (BD)

Cell population abundance

10000 cells were analyzed after removing cell debris.

Gating strategy

Cell debris was excluded using a FSC-A vs SSC-A gate and the aggregates were excluded via a SSC-H vs SSC-A. The unstained cells were gated as the negative control and PAX6 stained cells or NESTIN stained cells were used as the single positive control.

☒ Tick this box to confirm that a figure exemplifying the gating strategy is provided in the Supplementary Information.
